# Methylation‐Induced Permanent Charge Polarization in Covalent Organic Frameworks for Visible Light‐Driven Water Decontamination and Disinfection

**DOI:** 10.1002/advs.202520563

**Published:** 2026-01-04

**Authors:** Xuewen Peng, Huaji Pang, Niu Feng, Dekang Huang, Chunpeng Jiao, Jingbin Zeng, Yonggang Xiang, Yiping Chen

**Affiliations:** ^1^ College of Food Science and Technology Huazhong Agricultural University Wuhan 430070 China; ^2^ State Key Laboratory of Marine Food Processing and Safety Control Dalian Polytechnic University Dalian 116034 China; ^3^ College of Chemistry Huazhong Agricultural University Wuhan 430070 China; ^4^ PhiChem Corporation Shanghai 201900 China; ^5^ College of Chemistry and Chemical Engineering and State Key Laboratory of Heavy Oil Processing China University of Petroleum (East China) Qingdao 266580 China

**Keywords:** antibiotic residues, covalent organic frameworks, photocatalysis, reactive oxygen species

## Abstract

Covalent organic frameworks (COFs) show promise for photocatalytic environmental remediation and antibacterial applications; however, their efficiency is often constrained by strong excitonic effects that impede charge separation. Here, a targeted in situ methylation strategy is reported to engineer permanent cationic centers within a robust non‐substituted quinoline‐linked COF (NQ‐COF_S1_). Methyl grafting at the nitrogen sites generates quaternary ammonium groups, inducing pronounced local charge polarization and a strong built‐in electric field. This modification drastically reduces the exciton binding energy from 41 to 33 meV, thereby promoting highly efficient charge separation. In synergy with electron‐rich thiophene units, the resulting NQ‐COF_S1_‐Me exhibits outstanding photocatalytic activity, characterized by strong reactive oxygen species generation. It achieves > 95% inactivation of Gram‐positive, Gram‐negative, and drug‐resistant bacteria within 10 min, and 96.84% degradation of chloramphenicol—38.72 times faster than NQ‐COF_S1_. These findings demonstrate that methylation‐induced permanent charge polarization offers a powerful strategy for developing high‐performance photocatalytic COFs with broad potential in environmental and public health applications.

## Graphical abstract

A targeted in situ methylation strategy is developed to engineer cationic centers in a quinoline‐linked COF (NQ‐COF_S1_‐Me), creating a strong built‐in electric field, which effectively suppresses excitonic effects and promotes charge separation. The optimized material demonstrates powerful photocatalytic ROS generation for rapid bacterial inactivation and efficient degradation of antibiotic pollutants.

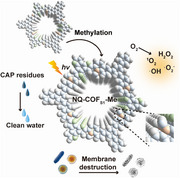

## 1 Introduction

The prevalence of emerging organic contaminants—including pharmaceuticals, personal care products, endocrine‐disrupting chemicals, and notably antibiotics—in aquatic environments has raised significant concern due to their potential risks to aquatic ecosystems and human health.^[^ ^1^, ^2^, ^3^, ^4^ ^]^ Antibiotic residues accelerate the development and spread of antibiotic resistance, posing a substantial threat by enabling the transmission of antibiotic‐resistant bacteria (ARB).^[^ ^5^ ^]^ ARB can rapidly proliferate in water bodies, and pathogenic strains may enter humans and cultured organisms through the food chain, thereby triggering epidemics and infections caused by untreatable “superbugs”.^[^ ^6^, ^7^, ^8^, ^9^, ^10^ ^]^ Therefore, developing effective methods for degrading antibiotics and controlling ARB is essential.^[^ ^11^, ^12^ ^]^ Photocatalytic technology, which harnesses inexhaustible solar energy to generate electron–hole pairs in semiconductor catalysts for driving redox reactions, offers a promising, low‐cost, and high‐efficiency strategy to address antibiotic pollution.^[^ ^13^, ^14^, ^15^, ^16^ ^]^ Furthermore, photocatalytic antibacterial therapy leverages the destructive effect of reactive oxygen species (ROS), such as hydroxyl radicals (•OH) and superoxide radicals (•O_2_^−^), presenting a highly promising non‐antibiotic antimicrobial approach.^[^ ^17^, ^18^, ^19^ ^]^ This technique offers precise spatiotemporal control, substrate versatility, non‐invasiveness, and a reduced likelihood of inducing resistance. Collectively, photocatalytic technology demonstrates considerable potential as a powerful tool for environmental remediation and public health protection.

Photosensitizers, such as silver, TiO_2_, copper, and zinc oxide nanoparticles, are the critical factor in determining photocatalytic performance.^[^ ^20^, ^21^, ^22^, ^23^, ^24^, ^25^, ^26^, ^27^ ^]^ However, these conventional photosensitizers may suffer from several drawbacks: a narrow range of UV absorption, leakage of toxic metal ions, and loss of active ingredients during application due to the photo‐corrosion effect. Reticular framework materials, known for their structural tailorability and ease of pore functionalization, are well‐suited as ideal photocatalysts.^[^ ^28^, ^29^ ^]^ Hydrogen‐bonded organic frameworks (HOFs) form their structures via hydrogen‐bonding interactions.^[^ ^30^, ^31^ ^]^ This relatively “soft” connection mode endows HOFs with unique adaptability. However, the topology of HOFs is sustained by weak hydrogen bonds, which often result in structural instability.^[^ ^32^ ^]^ In contrast, novel supramolecular organic frameworks (SOFs) rely on supramolecular interactions such as *π–π* stacking and van der Waals forces to assemble their frameworks.^[^ ^33^ ^]^ These weak, dynamic interactions allow SOFs to undergo reversible structural transformations under external stimuli, imparting excellent stimulus‐responsiveness.^[^ ^34^ ^]^ This property makes SOFs particularly suitable for applications such as smart sensors and controlled drug‐release systems, rather than serving as photosensitizers.^[^ ^35^ ^]^ Metal–organic frameworks (MOFs), constructed from organic linkers and transition‐metal nodes, are attractive due to their atomically dispersed metal sites.^[^ ^36^, ^37^, ^38^, ^39^ ^]^ However, most MOFs still suffer from their instability and can deteriorate under humid conditions, which limits their repeated use.^[^ ^40^, ^41^ ^]^

Covalent organic frameworks (COFs) are promising alternatives due to their excellent optical properties and chemical stability.^[^ ^42^, ^43^, ^44^, ^45^, ^46^, ^47^ ^]^ Besides, certain COFs display semiconductor properties, functioning as type I‐like photosensitizers that enable electron–hole pair separation.^[^ ^48^, ^49^ ^]^ Nevertheless, the low dielectric properties of COFs often lead to strong excitonic effects (the Coulomb interaction between photoinduced electrons and holes), which hinder photocatalysis.^[^ ^50^, ^51^ ^]^ Local charge polarization has been found to significantly enhance carrier charge separation in bulk photocatalysts by driving photogenerated carriers from the bulk to the target surface reaction. Recently, single‐atom transition‐metal sites (e.g., Ir and Ru) anchored on sp2c‐linked COF (sp2c‐COF) skeletons were developed by Wu et al. to enhance photocatalytic activity.^[^ ^52^ ^]^ However, the scarcity and high cost of rare metals restrict large‐scale production and practical application. Oxazole/thiazole‐linked COFs, synthesized via a one‐pot strategy by Hou et al., can effectively modulate exciton (e^−^–h^+^ pair) dissociation in COFs by adjusting the π‐conjugation and local charge polarization of the skeleton, thus enabling exceptional degradation performance of paracetamol under visible‐light irradiation.^[^ ^53^ ^]^ Notably, while imine‐to‐thiazole linkage conversion increases rigidity, the lower bond energies of C─O/C─S bonds render these COFs less stable than non‐substituted quinoline (NQ) ‐linked systems, in which conjugated linkages provide superior chemical resilience and retain photocatalytic effects in complex matrices.^[^ ^54^ ^]^

Therefore, benzo[1,2‐b:3,4‐b“:5,6‐b”']trithiophene‐2,5,8‐tricarbaldehyde and 1,3,5‐tris(4‐aminophenyl)benzene were employed as building blocks to construct an ultrastable NQ‐linked COF (NQ‐COF_S1_) via a one‐pot synthesis. To address the persistent challenge of exciton dissociation in COFs, a targeted methylation strategy was implemented by grafting methyl groups (Me) onto the nitrogen sites of quinoline linkages, generating permanent cationic centers (quaternary ammonium groups) and forming the charged framework NQ‐COF_S1_‐Me. This critical modification resulted in a substantially reduced exciton binding energy (E_b_ = 33 meV) and pronounced charge polarization, which synergized with the inherent thiophene‐sulfur units to drastically enhance photocatalytic activity. Under visible light, NQ‐COF_S1_‐Me exhibited exceptional multifunctional performance: 1) a significant increase in the generation of diverse ROS, including •OH, •O_2−_, singlet oxygen (^1^O_2_), hydrogen peroxide (H_2_O_2_); 2) rapid inactivation (> 95% within 10 min) of Gram‐negative, Gram‐positive, and drug‐resistant bacteria; 3) markedly enhanced antibiotic degradation, exemplified by a 38.72‐fold and 7.15‐fold increase in the degradation rate of chloramphenicol (CAP) compared to NQ‐COF_S1_ and a non‐thiophene methylated analogue NQ‐COF_A1_‐Me, respectively. This methylation strategy thus confers NQ‐COF_S1_‐Me with superior photocatalytic capabilities, positioning it as a transformative candidate for environmental remediation and public health protection.

## 2 Results and Discussion

### 2.1 The Synthesis and Characterization of NQ‐COF_S1_ and NQ‐COF_S1_‐Me

**Figure 1:**
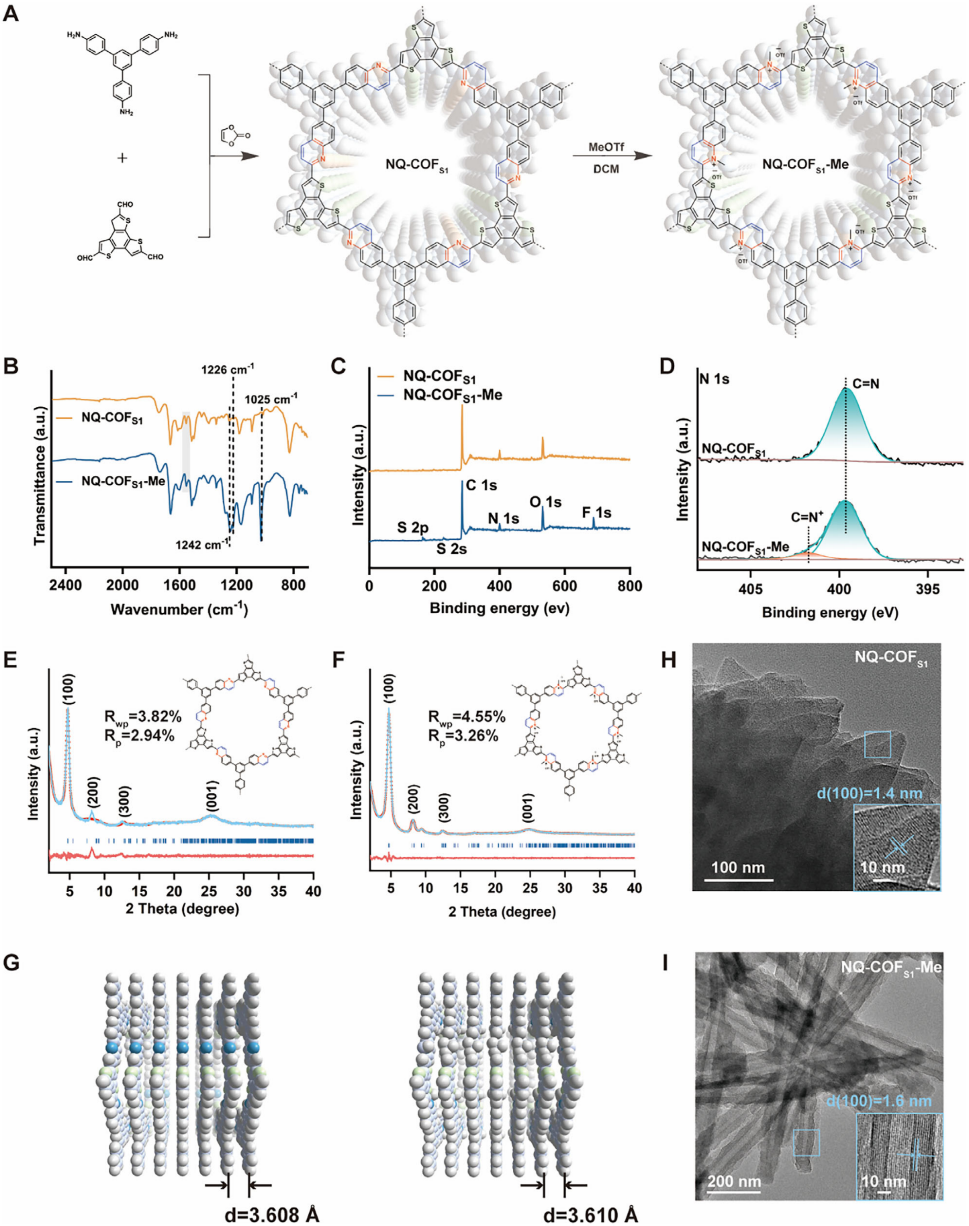
Characterization of NQ‐COF_S1_ and NQ‐COF_S1_‐Me. A) Schematic illustration of the synthesis of NQ‐COF_S1_‐Me by a two‐step method; B) FT‐IR spectra of NQ‐COF_S1_ and NQ‐COF_S1_‐Me; C) XPS survey spectra, and D) high‐resolution N 1s XPS spectra of NQ‐COF_S1_ and NQ‐COF_S1_‐Me; Pawley refinements of NQ‐COF_S1_ (E) and NQ‐COF_S1_‐Me (F) against their experimental PXRD patterns; G) Modeling of NQ‐COF_S1_ and NQ‐COF_S1_‐Me in eclipsed mode (side view). High‐resolution TEM image and crystal lattice spacing of H) NQ‐COF_S1_ and I) NQ‐COF_S1_‐Me.

As illustrated in **Figure** **1**A, using our recently reported one‐pot synthesis strategy, NQ‐COF_S1_ was successfully synthesized from benzo[1,2‐b:3,4‐b“:5,6‐b”']trithiophene‐2,5,8‐tricarbaldehyde and 1,3,5‐tris(4‐aminophenyl)benzene as building blocks. To verify the ionization of the quinoline structure in NQ‐COF_S1_, 2‐phenylquinoline was selected as a model substrate. Methyl trifluoromethanesulfonate was used as the ionization reagent, and the reaction was performed in dichloromethane at room temperature under an argon atmosphere for 12 h, yielding 1‐methyl‐2‐phenylquinoline with an isolated yield of 93%. The chemical structure of 1‐methyl‐2‐phenylquinoline was confirmed by nuclear magnetic resonance (NMR) spectroscopy (Figure S1, Supporting Information).

The chemical composition of NQ‐COF_S1_‐Me was characterized by Fourier‐transform infrared (FT‐IR) spectroscopy and X‐ray photoelectron spectroscopy (XPS). The FT‐IR spectrum of NQ‐COF_S1_ and NQ‐COF_S1_‐Me displays the characteristic band at 1514 cm^−1^ (gray shadow areas), confirming the successful construction of NQ linkages. Also, two signals at 1169 and 1341 cm^−1^ are attributed to sulfone O═S═O stretching bands.^[^ ^55^ ^]^ Compared to NQ‐COF_S1_, the FT‐IR spectra of NQ‐COF_S1_‐Me reveal a prominent peak at 1226 cm^−1^, attributed to the C─N methyl stretching vibration.^[^ ^56^ ^]^ The introduction of methyl groups strengthens the C─N stretching and C─H rocking vibrations of the NQ unit, resulting in an additional significant peak at 1242 and 1025 cm^−1^ (Figure 1B). These spectral features are consistent with those of the model compound 1‐methyl‐2‐phenylquinoline (Figure S2, Supporting Information). XPS measurements provided further insight into the methylation degree of NQ‐COF_S1_‐Me. In the survey XPS spectra of NQ‐COF_S1_ and NQ‐COF_S1_‐Me, in addition to C, N, and O, elements of F and S originating from OTF^−^ counterions were also detected in NQ‐COF_S1_‐Me (Figure 1C), supporting successful ionization. The high‐resolution N 1s XPS spectrum of NQ‐COF_S1_‐Me displays two distinct peaks at 399.8 and 401.8 eV, corresponding to the C═N bond of quinoline and the newly formed methyl C–N^+^, respectively (Figure 1D). The highly charged state of NQ‐COF_S1_‐Me was further confirmed by zeta potential analysis, with NQ‐COF_S1_‐Me exhibiting a much higher absolute zeta potential than NQ‐COF_S1_ (Figure S3, Supporting Information).

Static water contact angle measurements were used to evaluate hydrophilicity. According to Figure S4 (Supporting Information), the water contact angle of NQ‐COF_S1_‐Me was ≈62.45°, compared to 78.72° for NQ‐COF_S1_, indicating increased hydrophilicity due to the charged framework of NQ‐COF_S1_‐Me. The dispersion stability of NQ‐COF_S1_ and NQ‐COF_S1_‐Me in aqueous solution was evaluated (Figure S5, Supporting Information). Without ultrasonic treatment, NQ‐COF_S1_ aggregated significantly and settled at the bottom of the vial. In contrast, NQ‐COF_S1_‐Me demonstrated excellent dispersion stability, forming a homogeneous brown solution.

The crystal structure of NQ‐COF_S1_‐Me was examined by powder X‐ray diffraction (PXRD). As shown in Figure 1E,F, both NQ‐COF_S1_ and NQ‐COF_S1_‐Me exhibited strong diffraction peaks, indicating that the crystal structure is retained after methylation. NQ‐COF_S1_‐Me exhibited three distinct diffraction peaks at 4.69°, 8.19°, and 12.49°, assigned to the (100), (200), and (300) crystal planes, respectively. In comparison, NQ‐COF_S1_ showed peaks at 4.68°, 8.15°, and 12.44°, indicating a slight shift due to the reduced pore size following methylation. Using Materials Studio, AA‐stacked structure models of NQ‐COF_S1_ and NQ‐COF_S1_‐Me were constructed in the *P1* space group. The optimized cell parameters for both NQ‐COF_S1_ and NQ‐COF_S1_‐Me were a = b = 32.51 Å, c = 3.61 Å, α = β = 90°, γ = 120°, closely matching the theoretical values of the zigzag AA stacking model (Figure 1G). The simulated PXRD patterns agreed well with the experimental data, indicating that both materials preferentially adopt an AA‐stacked structure. Pawley refinement provided further confirmation, with the consistency factors as follows: NQ‐COF_S1_, Rwp = 3.82%, Rp = 2.94%; NQ‐COF_S1_‐Me, Rwp = 4.55%, Rp = 3.26%. As shown in Figure S6A,B (Supporting Information), N_2_ adsorption–desorption isotherms indicate Brunauer–Emmett–Teller (BET) surface areas of 463.72 and 96.44 m^2^ g^−1^ for NQ‐COF_S1_ and NQ‐COF_S1_‐Me, respectively. Non‐local density functional theory analysis of the pore size distribution (Figure S6C,D, Supporting Information) reveals that both materials exhibit well‐defined pores, centered predominantly at 4.02 nm for NQ‐COF_S1_ and 3.80 nm for NQ‐COF_S1_‐Me. This result confirms a reduction in pore size following modification of NQ‐COF_S1_. Therefore, the combined experimental and simulated PXRD patterns, together with the N_2_ adsorption–desorption data, strongly support the successful formation of Methyl‐grafted NQ‐COF_S1_‐Me.

Scanning electron microscopy (SEM) images show that NQ‐COF_S1_ and NQ‐COF_S1_‐Me both exhibits a rod‐like structure at the micron scale (Figure S7, Supporting Information). High‐resolution transmission electron microscopy (HRTEM) images further reveal highly ordered crystal structures for both materials. Clear lattice fringes are observed, with lattice spacings of 1.8 nm for NQ‐COF_S1_ and 1.6 nm for NQ‐COF_S1_‐Me, corresponding to the (100) crystal planes (Figure 1H,I).

### 2.2 The Photophysical Properties of NQ‐COF_S1_ and NQ‐COF_S1_‐Me

**Figure 2:**
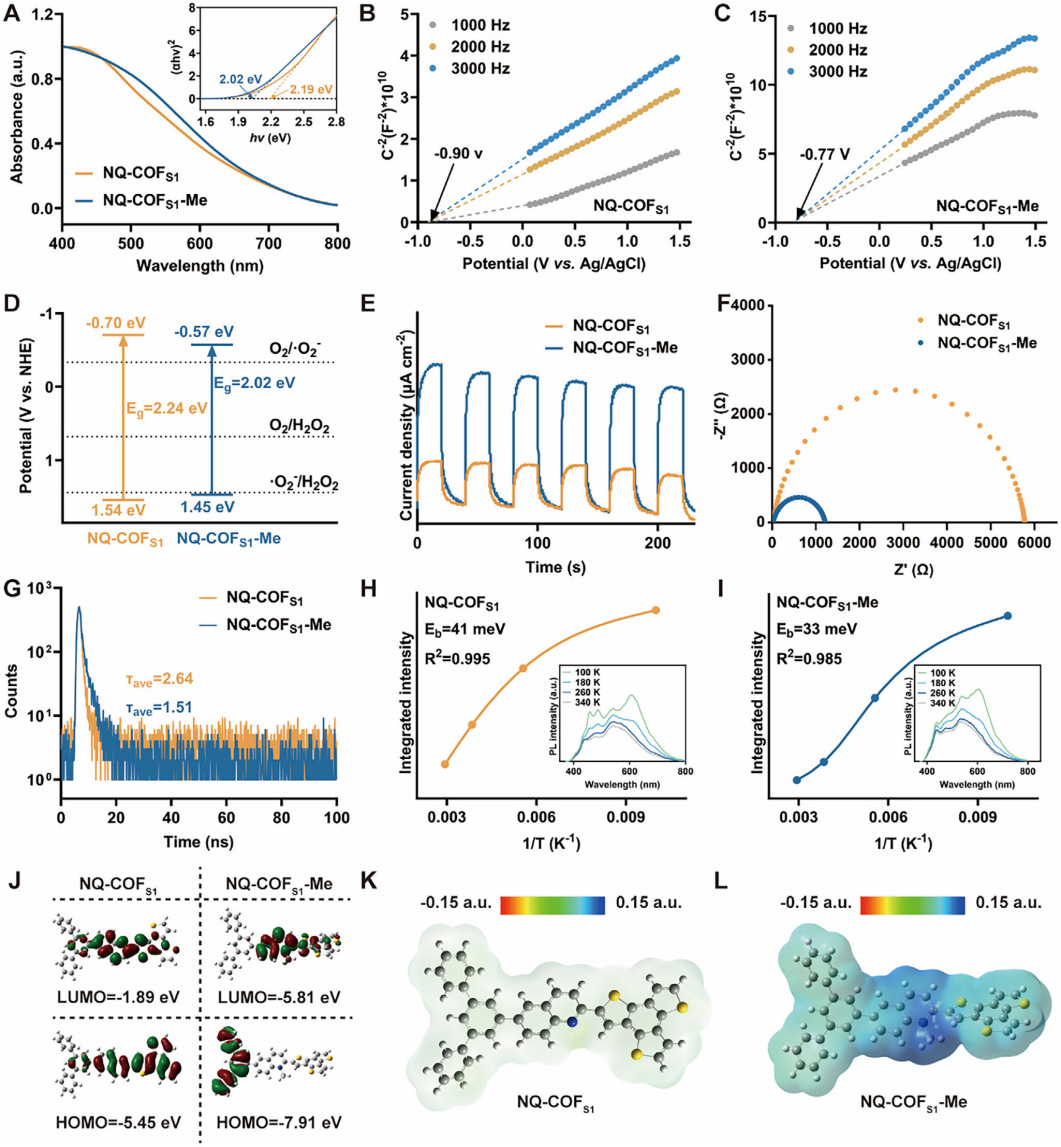
The photophysical properties of NQ‐COF_S1_ and NQ‐COF_S1_‐Me. A) UV−vis diffuse reflectance spectrum of NQ‐COF_S1_ and NQ‐COF_S1_‐Me. Insert: Tauc plot. Mott–Schottky plots of B) NQ‐COF_S1_ and C) NQ‐COF_S1_‐Me. D) Energy band positions, E) Transient photocurrents of, and F) EIS plots of NQ‐COF_S1_ and NQ‐COF_S1_‐Me. G) RPL decay curves of NQ‐COF_S1_ and NQ‐COF_S1_‐Me. Steady‐state PL spectra with a function of the reciprocal temperature of H) NQ‐COF_S1_ and I) NQ‐COF_S1_‐Me. Insert: Temperature‐dependent PL spectra. J) HOMO and LUMO diagrams. Electrostatic potential distribution of K) NQ‐COF_S1_ and L) NQ‐COF_S1_‐Me, red and blue colors represented electron accumulation and depletion.

The photophysical properties dictating the photocatalytic activity of COFs were systematically investigated using UV–vis diffuse reflectance spectroscopy (UV–vis DRS), electrochemical measurements, and photoluminescence (PL) spectroscopy. The optical bandgap (Eg) was first determined to evaluate visible‐light absorption capabilities. As shown in **Figure** **2**A, NQ‐COF_S1_‐Me displayed an absorption onset at 735.6 nm, representing a significant redshift compared to NQ‐COF_S1_ (703.4 nm). This shift signifies enhanced visible‐light utilization and superior light‐harvesting capacity for NQ‐COF_S1_‐Me. The Kubelka–Munk‐transformed reflectance data yielded an E_g_ of 2.02 eV for NQ‐COF_S1_‐Me, which is narrower than the 2.19 eV for NQ‐COF_S1_. To further characterize the band structure, Mott–Schottky (M‐S) measurements were performed at 1000, 2000, and 3000 Hz. The conduction band positions (E_CB_) for NQ‐COF_S1_ and NQ‐COF_S1_‐Me were −0.90 and −0.77 V (vs Ag/AgCl, respectively (Figure 2B,C). Converted to the normal hydrogen electrode (NHE), these correspond to −0.70 and −0.57 V (vs NHE. The valence band positions (E_VB_) were calculated from E_CB_ = E_VB_ – E_g_, yielding values of 1.54 and 1.45 V (vs NHE) for NQ‐COF_S1_ and NQ‐COF_S1_‐Me, respectively. Notably, the E_CB_ of NQ‐COF_S1_‐Me (−0.57 V vs NHE) is considerably more negative than the potential required for O_2_ reduction to •O_2_^−^ (−0.33 V vs NHE) and sufficient for subsequent conversion to H_2_O_2_ (H_2_O_2_/O_2_ = 0.68 V vs NHE), supporting the feasibility of these processes (Figure 2D).^[^ ^57^, ^58^ ^]^ Electrochemical impedance spectroscopy (EIS) and photocurrent response measurements provided additional insight into the photocatalytic behavior. NQ‐COF_S1_‐Me exhibited significantly higher photocurrent density compared to NQ‐COF_S1_, demonstrating more efficient charge separation and reduced recombination (Figure 2E). Consistently, methyl modification substantially decreased the charge transfer resistance from 5749.4 Ω (NQ‐COF_S1_) to 1190.7 Ω (NQ‐COF_S1_‐Me) (Figure 2F). Exciton separation efficiency was evaluated using steady‐state and time‐resolved photoluminescence (TRPL) spectroscopy. Steady‐state PL measurements revealed substantially lower emission intensity for NQ‐COF_S1_‐Me than for NQ‐COF_S1_ (Figure S8, Supporting Information). Time‐resolved PL emission spectra indicated a shorter average lifetime for NQ‐COF_S1_‐Me (1.51 ns) compared to NQ‐COF_S1_ (2.64 ns) (Figure 2G). The reduced PL intensity and shorter lifetime confirm enhanced charge separation and utilization efficiency in NQ‐COF_S1_‐Me, attributable to the combined effects of a charged skeleton and electron regulation induced by methyl modification. To further investigate the effect of methyl modification on exciton dissociation, the exciton binding energy (E_b_) was determined from temperature‐dependent PL spectra. E_b_ represents the Coulombic interaction binding photogenerated electron–hole pairs and dictates exciton dissociation kinetics. The PL peak intensities of both NQ‐COF_S1_ and NQ‐COF_S1_‐Me increased as the temperature decreased from 340 K to 100 K, consistent with thermally activated nonradiative recombination processes (Figure 2H,I).^[^ ^59^ ^]^ E_b_ values were obtained by nonlinear fitting using the equation I(T) = I_0_/ (1 + Ae(−E_b_/kB/T)).^[^ ^60^ ^]^ NQ‐COF_S1_ exhibited an E_b_ of 41 meV, whereas NQ‐COF_S1_‐Me exhibited a reduced E_b_ of 33 meV. A lower E_b_ facilitates exciton dissociation into long‐lived free charge carriers,^[^ ^61^ ^]^ indicating that excitons in NQ‐COF_S1_‐Me can more readily overcome thermodynamic barriers and dissociate into free electrons and holes at this temperature. These findings demonstrate that methylation promotes exciton dissociation in NQ‐COF_S1_‐Me, enhancing the migration of photogenerated electrons from the bulk to the material surface for interfacial catalytic reactions.

Density functional theory (DFT) simulations were performed on representative fragments of NQ‐COF_S1_ and NQ‐COF_S1_‐Me to elucidate the mechanism of methyl‐promoted intramolecular excited electron transfer. As shown in Figure 2J, NQ‐COF_S1_ exhibits uniform delocalization of the highest occupied molecular orbital (HOMO) and lowest unoccupied molecular orbital (LUMO), whereas NQ‐COF_S1_‐Me displays marked spatial separation between the HOMO and LUMO, accompanied by a reduced energy gap. This computational observation aligns with the experimental photophysical data, supporting enhanced electron affinity in the methylated framework. Electrostatic potential (ESP) analysis further revealed notable electron density reorganization upon methyl modification (Figure 2K,L). In NQ‐COF_S1_, the quinoline N atom carries a lone pair, generating a localized region of negative electrostatic potential. This site readily captures holes, promoting electron–hole complex formation and limiting electron transfer. By contrast, Methyl substitution converts this N atom into a quaternary ammonium group, introducing a permanent positive charge and markedly increasing electrostatic potential fluctuations across the framework. This modification produces local charge separation and a strong, localized electric field, thereby optimizing migration pathways for photogenerated carriers and further enhancing photocatalytic activity.

### 2.3 Photo–Driven ROS Generation of COFs

**Figure 3:**
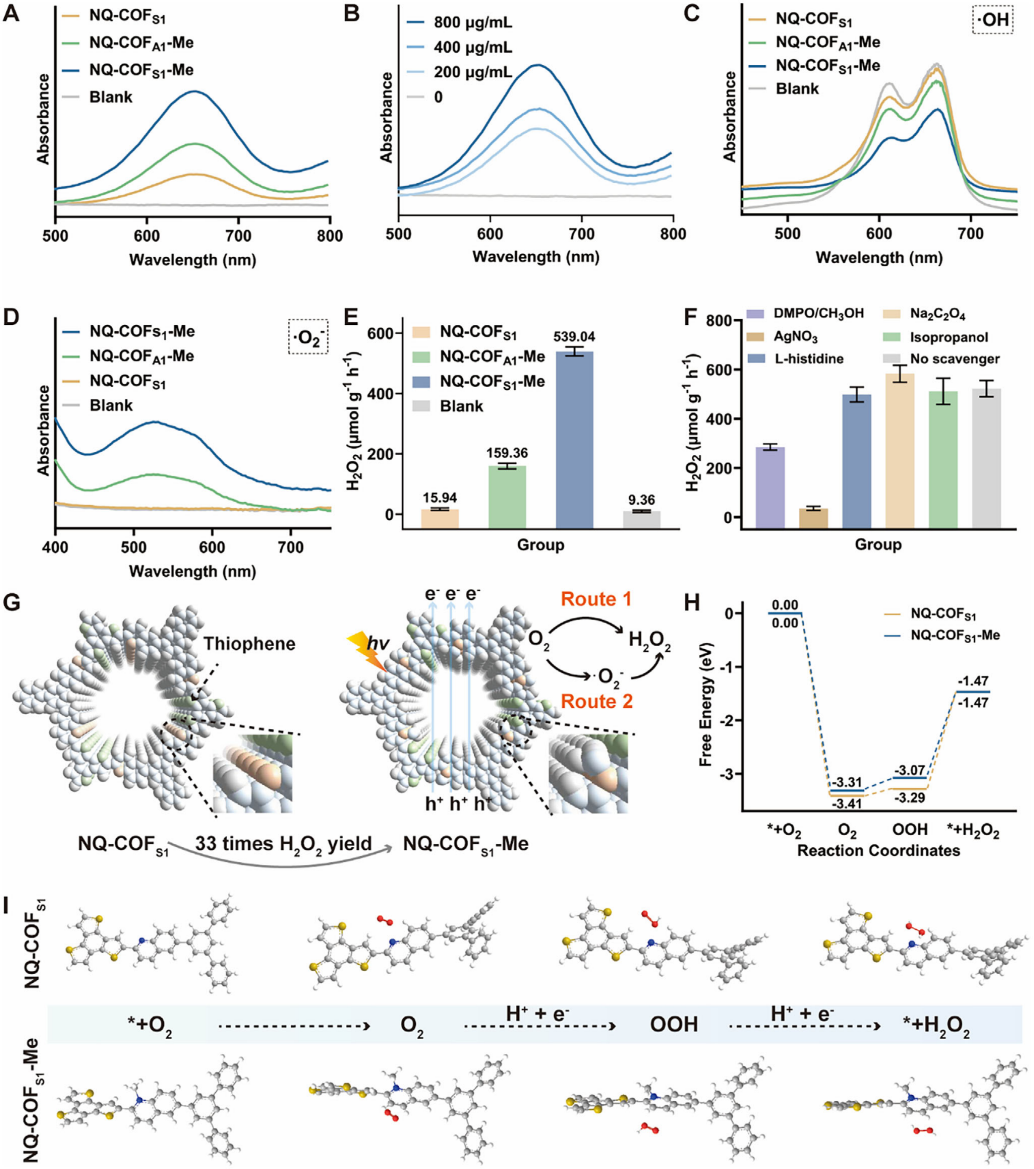
Light–Driven ROS Generation of COFs. A) Absorption spectra of TMB probe for ROS generation induced by different COFs. B) Absorption spectra of TMB probe for ROS generation induced by different concentrations of NQ‐COF_S1_‐Me. C) MB solution degradation curves for detecting the generation of •OH. D) Absorption spectra of NBT for detecting the generation of •O^2−^. E) The H_2_O_2_ production evaluation induced by different COFs. F) Effect of scavengers on the performance of NQ‐COF_S1_‐Me photocatalytic generation of H_2_O_2_. G) Schematic of H_2_O_2_ generation for the NQ‐COF_S1_‐Me under light irradiation. H) The Gibbs free energy profiles for photocatalytic H_2_O_2_ evolution reactions over NQ‐COF_S1_ and NQ‐COF_S1_‐Me. I) The Gibbs free energies of intermediates generated at different steps for NQ‐COF_S1_ and NQ‐COF_S1_‐Me. The error bars represent mean ± SD from triplicate replications (n = 3).

Building on the enhanced carrier separation efficiency, the photocatalytic ROS generation performance of NQ‐COF_S1_‐Me was systematically evaluated. To clarify the specific contribution of the thiophene structure, the non‐thiophene analog NQ‐COF_A1_‐Me was used as a control (Figure S9, Supporting Information). Colorimetric assays under light irradiation revealed clear differences in ROS production profiles. All materials (NQ‐COF_S1_, NQ‐COF_A1_‐Me, NQ‐COF_S1_‐Me) catalyzed the oxidation of the 3,3′,5,5′‐tetramethylbenzidine (TMB) probe, resulting in characteristic absorption at 650 nm, with NQ‐COF_S1_‐Me displaying the most intense signal (**Figure** **3**A). Concentration‐dependent experiments confirmed that NQ‐COF_S1_‐Me exhibited superior activity, as evidenced by a progressive increase in absorbance at 650 nm with higher catalyst loading (Figure 3B). Assessment of •OH production via methylene blue (MB) degradation indicated substantially enhanced MB decomposition for NQ‐COF_S1_‐Me, approximately twice that observed for NQ‐COF_A1_‐Me and significantly greater than NQ‐COF_S1_, indicating robust •OH generation (Figure 3C). The nitro blue tetrazolium (NBT) assay for •O_2_^−^, monitored at 530 nm, showed minimal change for NQ‐COF_S1_, but a marked increase in monoformazan formation for NQ‐COF_S1_‐Me, which was roughly double that of NQ‐COF_A1_‐Me (Figure 3D). Electron paramagnetic resonance (EPR) spectroscopy further confirmed efficient ROS generation, including •OH, •O_2_^−,^ and ^1^O_2_. With NQ‐COF_S1_‐Me producing the strongest •OH, •O_2_^−^ and ^1^O_2_ signal under xenon lamp illumination, followed by NQ‐COF_A1_‐Me and NQ‐COF_S1_ (Figure S10, Supporting Information). The photocatalytic H_2_O_2_ production of the COFs was quantified using a potassium iodide/potassium hydrogen phthalate (KI‐C_8_H_5_KO_4_) assay under white light. As shown in Figures 3E and S11 (Supporting Information), NQ‐COF_S1_‐Me exhibited superior H_2_O_2_ evolution (539.04 µmol g^−1^ h^−1^), exceeding NQ‐COF_A1_‐Me and NQ‐COF_S1_ by factors of 3.38 and 33, respectively.

Since H_2_O_2_ production is intimately linked to the generation of other ROS, studying H_2_O_2_ formation can elucidate ROS production mechanisms. Band alignment analysis supports the exclusive oxygen reduction reaction (ORR) pathway for H_2_O_2_ formation: NQ‐COF_S1_‐Me's energy levels facilitate the reductions O_2_ to •O_2_^−^ (−0.33 eV), O_2_ to H_2_O_2_ (0.68 eV), and •O_2_^−^ to H_2_O_2_ (1.44 eV).^[^ ^62^ ^]^ Surface methylation induces pronounced charge polarization in both NQ‐COF_A1_‐Me and NQ‐COF_S1_‐Me, thereby enhancing ORR kinetics. Importantly, thiophene units in NQ‐COF_S1_‐Me cooperatively enhance O_2_ adsorption and multielectron reduction. This synergistic effect accounts for the 3.38‐fold increase in H_2_O_2_ production relative to the non‐thiophene NQ‐COF_A1_‐Me. Atmosphere‐controlled experiments confirmed the ORR mechanism: H_2_O_2_ production was negligible under N_2_ (Figure S12, Supporting Information), establishing O_2_ as the essential reactant and ruling out alternative pathways. To elucidate the H_2_O_2_ formation mechanism, photocatalytic experiments with NQ‐COF_S1_‐Me were conducted in the presence of various scavengers: DMPO for •O_2_^−^, AgNO_3_ for e^−^, L‐histidine for ^1^O_2_, Na_2_C_2_O_4_ for h^+^, and isopropanol for •OH. As shown in Figure 3F, •O_2_^−^ scavenging (DMPO/CH_3_OH) strongly suppressed H_2_O_2_ generation, confirming •O_2_^−^ as a key intermediate. Electron scavenging (AgNO_3_) nearly eliminated H_2_O_2_ production, indicating that electrons are the primary drivers of O_2_ reduction. Moreover, H_2_O_2_ generation increased slightly with hole scavenging (Na_2_C_2_O_4_), possibly due to inhibition of electron–hole recombination. The negligible impact of •OH scavengers (isopropanol) on H_2_O_2_ production demonstrates that •OH are not primary intermediates in H_2_O_2_ formation, but rather secondary species derived from H_2_O_2_. Based on these findings, the pathway for H_2_O_2_ formation is as follows:

(1) $$O_{2}+2H^{+}+2e^{-}\to H_{2}O_{2}$$

(2) O2→∗O2−

(3) ∗O2−+H++e−→OOH∗

(4) $$\mathrm{OO}H^{*}+H^{+}+e^{-}\to \;H_{2}O_{2}$$

The H_2_O_2_ generation ability of NQ‐COF_S1_‐Me provides a good explanation for its excellent ROS production capacity. Under the light irradiation, the H_2_O_2_ was converted to •OH by further reaction with e.^−[^ ^63^, ^64^ ^]^ Although •O_2−_ can react with h⁺ to produce ^1^O_2_, the minimal impact of ^1^O_2_ quenchers on H_2_O_2_ production indicates that this redox pathway is not the dominant route for ^1^O_2_ generation (Figure 3F). Therefore, we suggest that the NQ‐COF_S1_‐Me excited by light to form e^−^ and h^+^. Those with strong Coulomb interactions bound to form neutral excitons on the singlet excited state would transfer to the lower‐energy triplet state through intersystem crossing, where the energy is transferred to the adsorbed O_2_ molecule to generate ^1^O_2_.^[^ ^65^ ^]^

To further elucidate the effect of methylation on H_2_O_2_ production by NQ‐COF_S1_‐Me, the changes in Gibbs free energy for the adsorption of intermediates at different sites were calculated by DFT (Figure 3H,I). Methyl modification significantly enhanced O_2_ adsorption capacity (from –3.31 to –3.41 eV) compared with NQ‐COF_S1_. Since ^*^OOH is a core intermediate in the two‐electron ORR pathway, the enhanced O_2_ adsorption capacity of NQ‐COF_S1_‐Me substantially promotes ^*^OOH formation, facilitating faster ORR kinetics and more efficient conversion of OOH to H_2_O_2_, ultimately leading to improved H_2_O_2_ production.

### 2.4 Photo‐Driven COF Activation for Pathogen Elimination

**Figure 4:**
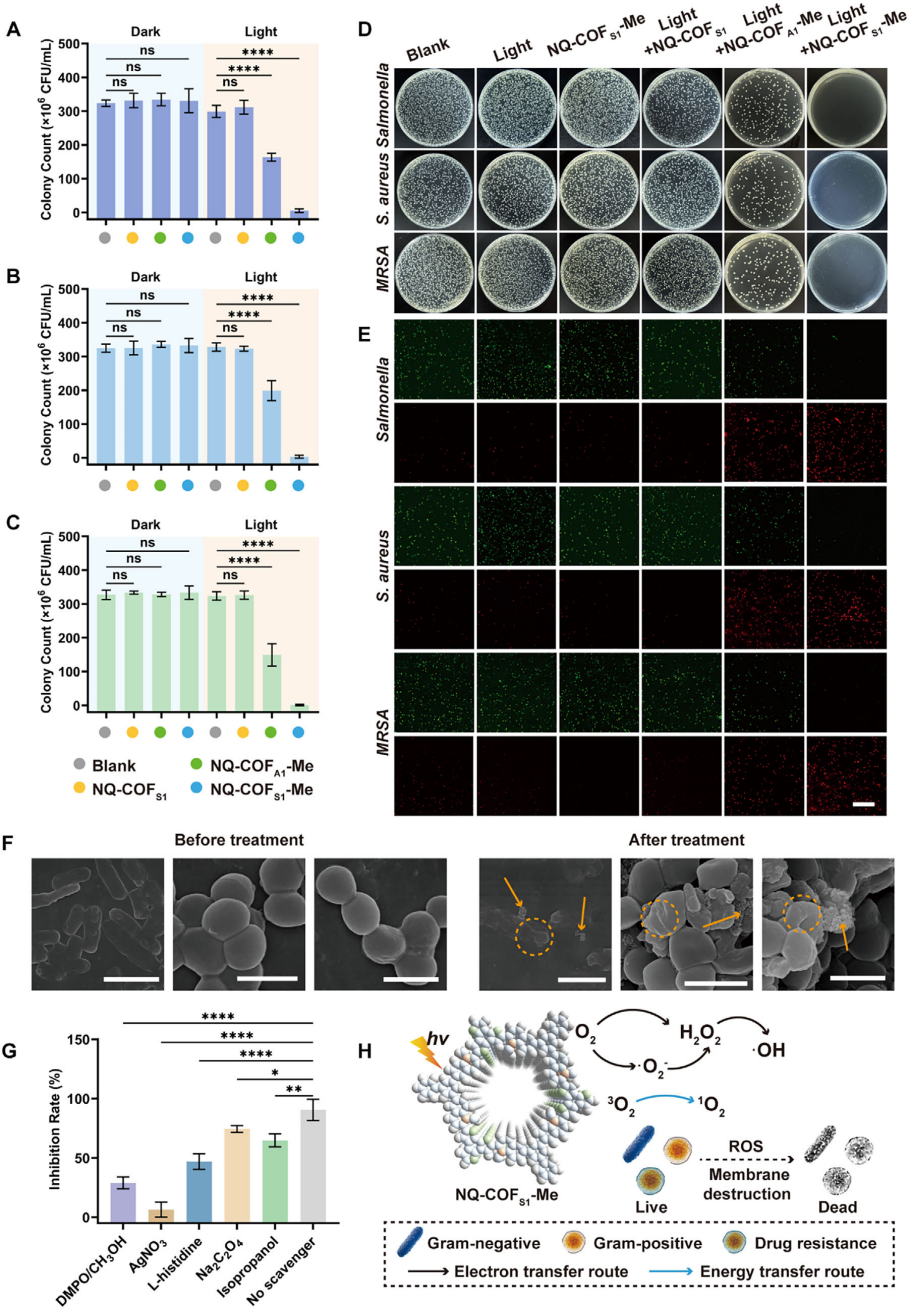
Photo‐driven COF activation for pathogen elimination. The colony count of A) *Salmonella*, B) *S. aureus*, and C) MRSA group of blank, NQ‐COF_S1_, NQ‐COF_A1_‐Me, and NQ‐COF_S1_‐Me exposed to white light or not. D) Spread‐plate images and E) CLSM images of LIVE/DEAD staining after inoculation with six different treatment groups. (Blank, Light, NQ‐COF_S1_‐Me, Light+NQ‐COF_S1_, Light+NQ‐COF_A1_‐Me, and Light+NQ‐COF_S1_‐Me). The scale bar represents 50 µm. F) SEM images of *Salmonella*, *S. aureus*, and MRSA before and after treatments. The scale bar represents 2 µm. G) Effect of scavengers on the performance of NQ‐COF_S1_‐Me photocatalytic inactivation of *S. aureus*. H) Schematic of ROS generation for the NQ‐COF_S1_‐Me photocatalytic inactivation of *S. aureus*. The error bars represent mean ± SD from triplicate replications (n = 3), with statistical significance indicated by asterisks (ns, not significant; ^*^ *p* < 0.05; ^**^ *p* < 0.01; ^***^ *p* < 0.001; ^****^ *p* < 0.0001).

Prompted by the exceptional photocatalytic ROS generation of NQ‐COF_S1_‐Me, the antibacterial efficacy of the COFs against clinically relevant pathogens—namely, Gram‐negative *Salmonella enterica* (*Salmonella*), Gram‐positive *Staphylococcus aureus* (*S. aureus*), and methicillin‐resistant *Staphylococcus aureus* (MRSA)—was systematically investigated. Viability assays performed under dark conditions revealed negligible reduction in colony‐forming units (CFU/mL) for all pathogens treated with NQ‐COF_S1_, NQ‐COF_A1_‐Me, or NQ‐COF_S1_‐Me (**Figure** **4**A–C), confirming their inherent biocompatibility and absence of dark toxicity. Under light irradiation, however, marked differences emerged: NQ‐COF_S1_ exhibited no significant bactericidal effect, NQ‐COF_A1_‐Me displayed moderate activity, and NQ‐COF_S1_‐Me achieved near‐complete eradication of all three pathogens, indicating its capacity as a potent broad‐spectrum photocatalytic bactericide.

These findings were further corroborated by spread‐plate assays and confocal laser scanning microscopy (CLSM) with LIVE/DEAD staining, in which dead bacteria are stained red and viable bacteria are stained green. Representative images of six treatment conditions—Blank, Light, NQ‐COF_S1_‐Me, Light+NQ‐COF_S1_, Light+NQ‐COF_A1_‐Me, and Light+NQ‐COF_S1_‐Me—are presented in Figures 4D,E and S13 (Supporting Information). The nearly complete transition to red fluorescence (dead cells) in the Light+NQ‐COF_S1_‐Me group is consistent with the CFU data, establishing that methyl–thiophene synergy is critical for potent photocatalytic disinfection.

The antibacterial efficacy of NQ‐COF_S1_‐Me is both dose‐ and time‐dependent. As shown in Figure S14 (Supporting Information), under the conditions of 10 min light irradiation and an NQ‐COF_S1_‐Me concentration of 384 µg mL^−1^, the inhibition rates against *Salmonella*, *S. aureus*, and MRSA reached 96.04%, 97.75%, and 97.46%, respectively. Notably, NQ‐COF_S1_‐Me retained outstanding antibacterial efficacy after 480 days of storage (Figure S15, Supporting Information), with inhibition rates of 94.53% for *Salmonella*, 95.75% for *S. aureus*, and 91.79% for *MRSA*, indicating minimal loss of activity. These results demonstrate that NQ‐COF_S1_‐Me displays robust sterilization capability against different types of bacteria and possesses excellent storage stability, attributes that are ascribed to the strong stability of the NQ linkage and persistent charge polarization conferred by methyl‐derived quaternary ammonium groups.

The mechanism underlying the antibacterial action of NQ‐COF_S1_‐Me was examined further. SEM images of bacteria before and after treatment with NQ‐COF_S1_‐Me revealed that, prior to treatment, *Salmonella* cells displayed sharp margins, and *S. aureus* and *MRSA* cells exhibited full and spherical morphologies. Following treatment, the blurred edges of *Salmonella* cells indicated compromised structural integrity, while *S. aureus* and *MRSA* cells showed pronounced wrinkling and deformation. In addition, extensive leakage of intracellular material was observed, indicating that NQ‐COF_S1_‐Me disrupts the cell membrane or cell wall structures, thereby leading to pathogen inactivation (Figure 4F).

To elucidate the contribution of different ROS, five specific scavengers were introduced: DMPO/CH_3_OH for •O_2_^−^, AgNO_3_ for electrons, isopropanol for •OH, L‐histidine for ^1^O_2_, and Na_2_C_2_O_4_ for holes. When *S. aureus* or *MRSA* was used as the model pathogen, the presence of scavengers for •O_2_^−^, ^1^O_2_, and •OH markedly impaired the photocatalytic antibacterial effect of NQ‐COF_S1_‐Me. Among them, •O_2_^−^ scavenging had the most pronounced effect, reflecting both its intrinsic cytotoxicity and its role as an essential intermediate for generating H_2_O_2_ and •OH. When electron scavengers were present, NQ‐COF_S1_‐Me exhibited minimal antibacterial activity, indicating that the absence of electrons severely restricts H_2_O_2_ and •O_2_^−^ production and, therefore, overall ROS generation (Figures 4G; S16A, Supporting Information). When *Salmonella* was used as the test organism, scavenging of •O_2_^−^ had a reduced effect, likely due to the moderate reactivity of •O_2_^−^ and the requirement for its conversion to more toxic ROS within the bacterial cell.^[^ ^66^ ^]^ The more complex and less permeable inner membrane of Gram‐negative bacteria further limits direct attack by •O_2_^−^ (Figure S16B, Supporting Information).^[^ ^67^ ^]^

Collectively, these results demonstrate that the high levels of localized, lethal ROS generated by the NQ‐COF_S1_‐Me photocatalyst efficiently damage pathogen cell membranes, yielding potent bactericidal effects against both Gram‐negative and Gram‐positive bacteria and addressing the challenge of bacterial drug resistance within a short period (Figure 4H).

To assess bactericidal efficacy under realistic conditions, the antibacterial performance of NQ‐COF_S1_‐Me was evaluated in various environmental water samples. As shown in Figure S17 (Supporting Information), NQ‐COF_S1_‐Me exhibited high antibacterial activity (> 91.5%) against *Salmonella*, *S. aureus*, and *MRSA* in diverse environments, including farm wastewater, lake water, and medical wastewater.

### 2.5 Photo‐Driven COF Activation for Pollutant Degradation

Pharmaceutical compounds are frequently detected in various aquatic environments due to their widespread use in human and veterinary medicine.^[^ ^68^ ^]^ Among these, antibiotic residues have attracted considerable attention due to their potential adverse effects. CAP, a broad‐spectrum antibiotic effective against Gram‐positive, Gram‐negative bacteria, and other microorganisms, is a particular concern.^[^ ^69^ ^]^ Conventional wastewater treatment plants are largely ineffective at removing CAP, raising concerns regarding the complete elimination of such antibiotic contaminants from water bodies. Given the outstanding photocatalytic properties of NQ‐COF_S1_‐Me, its potential for CAP degradation was systematically explored.

**Figure 5:**
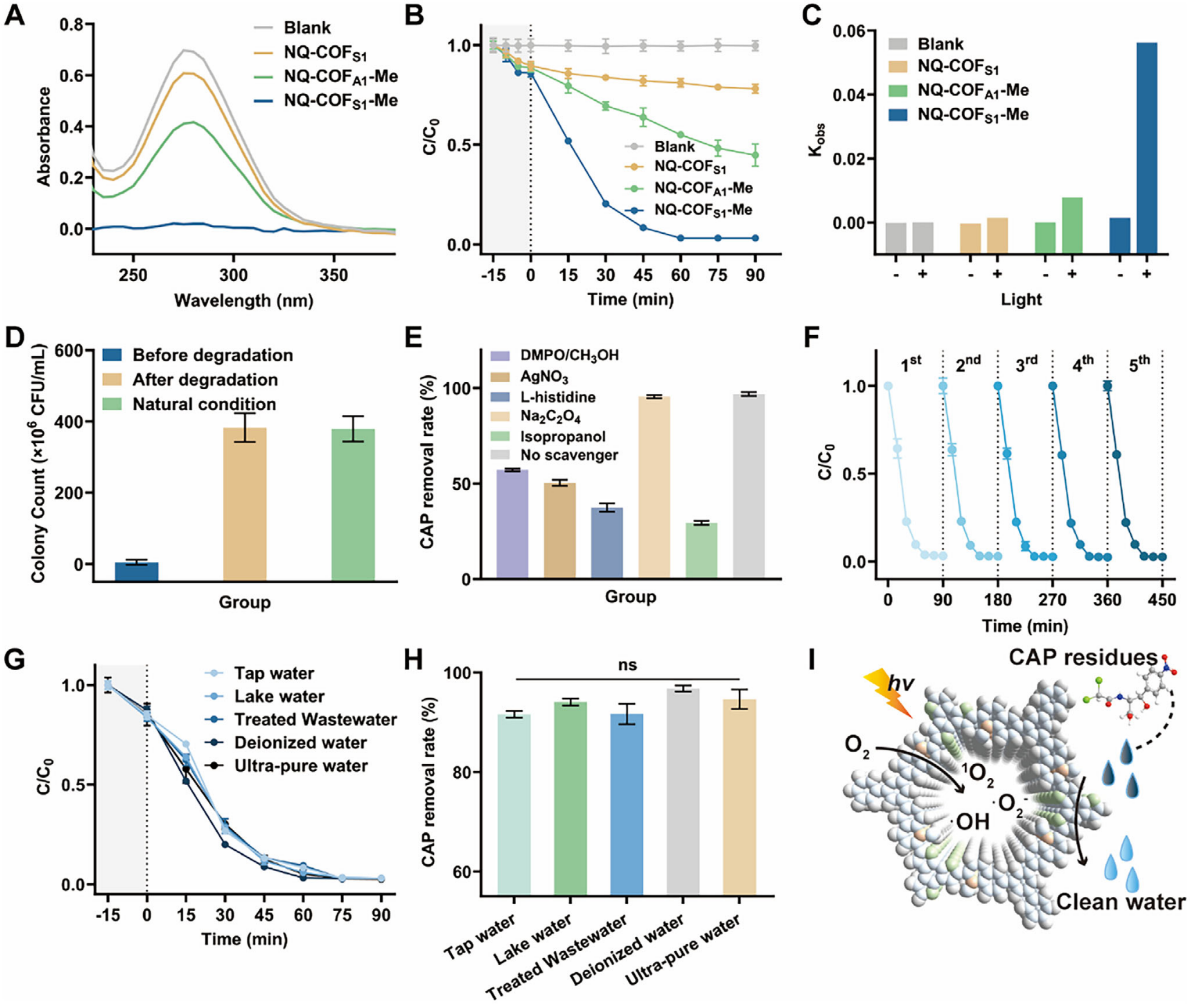
Photo‐driven COF activation for pollutant degradation. A) UV absorption spectrum of CAP solution after different COF photocatalytic degradation. B) CAP degradation efficiency under the various COFs treatment with irradiation. C) The corresponding kinetic rate constants under the various COFs for photocatalytic degradation. D) Colony count of before and after CAP degradation by NQ‐COF_S1_‐Me. E) Effect of scavengers on the performance of NQ‐COF_S1_‐Me photocatalytic degradation of CAP. F) 5 consecutive cycles of photocatalytic degradation performance evaluation. G) Effect of different water matrices on CAP degradation efficiency. H) Degradation percentage of CAP of NQ‐COF_S1_‐Me in different water matrices. I) Schematic of NQ‐COF_S1_‐Me photocatalytic degradation of CAP. The error bars represent mean ± SD from triplicate replications (n = 3), with statistical significance indicated by asterisks (ns, not significant; ^*^ *p* < 0.05; ^**^ *p* < 0.01; ^***^ *p* < 0.001; ^****^ *p* < 0.0001).

Initially, a proof‐of‐concept study was conducted using the synthesized COFs as catalysts for CAP treatment in aqueous environments. As shown in **Figure** **5**A, light irradiation induced a reduction in CAP concentration across all tested materials (NQ‐COF_S1_, NQ‐COF_A1_‐Me, and NQ‐COF_S1_‐Me), with NQ‐COF_S1_‐Me demonstrating the most significant removal. Increasing the concentration of COFs resulted in a pronounced decrease in CAP absorbance (C/C_0_), as evidenced in Figure S18 (Supporting Information). Control experiments performed under dark conditions (Figure S19, Supporting Information) yielded CAP removal rates below 17.24% within 90 min, attributable to adsorption by the porous COF framework, indicating negligible catalytic activation without visible light. In contrast, under light irradiation, NQ‐COF_S1_‐Me exhibited substantially higher CAP photodegradation rates than NQ‐COF_S1_ or NQ‐COF_A1_‐Me (Figure 5B). After 60 min, the CAP removal rates for NQ‐COF_S1_, NQ‐COF_A1_‐Me, and NQ‐COF_S1_‐Me were 18.99%, 45.02%, and 96.84%, respectively, highlighting the superior degradation capability of NQ‐COF_S1_‐Me.

Furthermore, as presented in Figures 5C and S20 (Supporting Information), the pseudo‐first‐order rate constant for NQ‐COF_S1_‐Me was 0.05629 min^−1^, representing a 38.72‐fold and 7.15‐fold enhancement over NQ‐COF_S1_ (0.001454 min^−1^) and NQ‐COF_A1_‐Me (0.00787 min^−1^), respectively. This substantial improvement highlights the effect of the thiophene moiety and the beneficial influence of methyl modification on CAP degradation kinetics. Importantly, the biotoxicity of CAP was completely eliminated following photocatalytic treatment. The original CAP solution displayed pronounced antibacterial activity, as indicated by low colony counts (Figure 5D), whereas the degraded solution exhibited virtually no inhibitory effect, confirming effective detoxification after treatment with NQ‐COF_S1_‐Me.

To elucidate the mechanism underlying CAP degradation by NQ‐COF_S1_‐Me, a series of controlled experiments was conducted with the addition of various scavengers (Figures 5E; S21, Supporting Information). The presence of DMPO/CH_3_OH led to a 39.78% decrease in degradation rate, suggesting a limited contribution of •O_2_^−^ in this system. In contrast, L‐histidine and isopropanol decreased the degradation rates by 59.44% and 67.39%, respectively, indicating that multiple reactive species contribute to CAP degradation, with •OH radicals serving as the primary ROS.

The reusability of NQ‐COF_S1_‐Me was also investigated. In recycling experiments, more than 97.01% of CAP was removed by NQ‐COF_S1_‐Me after five consecutive catalytic cycles (Figure 5F), demonstrating excellent catalytic durability. The effect of various ions on CAP degradation was also assessed. As shown in Figure S22 (Supporting Information), the addition of cations had negligible influence on adsorption and degradation, while Cl^−^ and CO_3_^2−^ exhibited slight inhibitory effects on the photocatalytic process, likely due to the interaction of h^+^ species with Cl^−^ and CO_3_.^2−[^ ^70^ ^]^ Finally, the degradation of CAP was evaluated in different natural water matrices, including tap water, lake water, treated wastewater, and deionized water (Figure 5G). Lake water minimally reduced CAP removal (by only 2.71%). Although there was a slight decrease in degradation efficiency in tap water and treated wastewater, high removal rates (91.60% and 91.87%, respectively) were still achieved within 60 min, with complete removal possible by prolonging the treatment time (Figure 5H). Collectively, NQ‐COF_S1_‐Me demonstrated excellent CAP degradation performance in a range of water environments and effectively converted toxic wastewater into non‐toxic effluent, supporting its practical application potential (Figure 5I).

In addition, to assess the broader application of NQ‐COF_S1_‐Me, its efficacy against other antibiotics—specifically sulfamethoxazole (SMX) and norfloxacin (NFX)—was evaluated. As shown in Figure S23 (Supporting Information), under light irradiation, NQ‐COF_S1_‐Me achieved nearly complete removal of SMX (97.89%) within 90 min and NFX (96.11%) within 120 min. While the rate constants for SMX (0.0016 min^−1^) and NFX (0.0008 min^−1^) were lower than that for CAP (Figure S24, Supporting Information), extended irradiation yielded full removal, indicating the versatility of NQ‐COF_S1_‐Me in degrading multiple antibiotic pollutants.

## 3 Conclusion

In summary, an ultrastable NQ‐COF with robust NQ linkages (NQ‐COF_S1_) was developed, and subsequent methyl substitution at the nitrogen site of the NQ linkage yielded NQ‐COF_S1_‐Me, which demonstrated markedly enhanced charge separation efficiency (E_b_ = 33 meV). Under light irradiation, NQ‐COF_S1_‐Me demonstrated the outstanding ability in ROS production. In addition, NQ‐COF_S1_‐Me displayed potent broad‐spectrum antibacterial activity, achieving rapid inactivation of Gram‐positive bacteria, Gram‐negative bacteria, and drug‐resistant strains within 10 min. Notably, NQ‐COF_S1_‐Me demonstrated robust degradation of CAP, with the degradation rate increased by 38.72‐fold compared with NQ‐COF_S1_. Moreover, this study investigated the effect of methyl grafting on the photocatalytic performance of COF materials, highlighting the synergistic enhancement of photocatalytic activity by methyl and thiophene groups, and providing new directions for the design of multifunctional photocatalytic materials in future applications.

## 4 Experimental Section

### Synthesis of NQ‐COF_S1_

A Pyrex glass tube (10 mL) was charged with benzo[1,2‐b:3,4‐b“:5,6‐b”']trithiophene‐2,5,8‐tricarbaldehyde (33 mg, 0.10 mmol), 1,3,5‐tris(4‐aminophenyl)benzene (35 mg, 0.10 mmol), 1,3‐dioxol‐2‐one (36 µL), MgSO_4_ (72 mg), [Cp*RhCl_2_]_2_ (5 mg), o‐dichlorobenzene (1.0 mL), and acetic acid (50 µL). Subsequently, the tube was sonicated for 10 min, degassed by three freeze‐pump‐thaw cycles (liquid nitrogen), and sealed under vacuum. After being heated in an oven at 120 °C for 3 days, the cooled suspension was centrifuged to separate the solid, which was repeatedly washed by tetrahydrofuran (THF), MeOH, and water until the solvent was colorless. The NQ‐COF_S1_ was finally obtained as a sepia powder after being dried under vacuum at 80 °C.

### Synthesis of NQ‐COF_S1_‐Me

To synthesize the NQ‐COF_S1_‐Me, a 50 mL round‐bottom flask was charged with the corresponding NQ‐COF_S1_ (33.7 mg), MeOTf (8.2 mg, 0.05 mmol), and DCM (10 mL). After being degassed with Ar for 10 min, the mixture was reacted at room temperature for 12 h. The solid was separated by filtration under reduced pressure, and washed with DCM, CH_3_OH, distilled water, and tetrahydrofuran (THF) successively. Finally, NQ‐COF_S1_‐Me (33.2 mg) was obtained as a dark brown powder after being dried under vacuum at 80 °C.

## Author Contributions

X.P., H.P., and N.F. contributed equally to this work. X.P., H.P., and N.F. were responsible for the conceptualization, methodology, data curation, and writing of the original draft. D.H., C.J., J.Z., and Y.X. were responsible for the investigation, writing, reviewing, and editing. Y.C. was responsible for formal analysis, supervision, and funding acquisition.

## Conflict of Interest

The authors declare no conflict of interest.

## Supporting information



Supporting Information

## Data Availability

The data that support the findings of this study are available from the corresponding author upon reasonable request.
