# Supplementary material for: Methylation‐Induced Permanent Charge Polarization in Covalent Organic Frameworks for Visible Light‐Driven Water Decontamination and Disinfection
Source: Adv Sci (Weinh). 2026 Jan 4;13(12):e20563. doi: 10.1002/advs.202520563 (PMC12948231; doi:10.1002/advs.202520563)
Supplement: Supplementary file 1 — Supporting Information [file ADVS-13-e20563-s001.docx]

Supporting Information

Methylation-Induced Permanent Charge Polarization in Covalent Organic Frameworks for Visible Light-Driven Water Decontamination and Disinfection

Xuewen Peng^#^, Huaji Pang^#^, Niu Feng^#^, Dekang Huang, Chunpeng Jiao, Jingbin Zeng^*^, Yonggang Xiang^*^ and Yiping Chen^*^

**1. Materials and methods**

**1.1 General information**

Unless otherwise stated, all the chemicals were purchased in analytical purity from commercial suppliers and used directly without further purification.

Powder X-ray diffraction (PXRD) patterns were collected on a Bruker D8 Advance diffractometer with Cu Kα radiation (2*θ* range: 2-40°; Scan step size: 0.02°; Time per step: 1 s). Fourier transform infrared (FT-IR) spectra were collected on a Nicolet 6700 spectrometer (Thermo Scientific, USA) equipped with an ATR cell. The specific Brunauer-Emmett-Teller (BET) surface area and pore size distribution were measured using a Micrometrics ASAP 2040 instrument at 77 K. High resolution transmission electron microscope (HRTEM) images were obtained on a Talos F200x instrument at an accelerating voltage of 200 kV. Scanning electron microscopy (SEM) images were collected using a Hitachi SU 8010 microscope. X-ray photoelectron spectroscopy (XPS) measurements were performed on a Thermo ESCALAB 250 spectrometer with non-monochromatic Al Kα x-rays as the excitation source and C 1s (284.4 eV) as the reference line. Solid-state diffuse reflectance Ultraviolet–visible spectroscopy (UV-vis) spectra were collected on a Shimadzu UV 3600 Spectrophotometer with BaSO_4_ as the reference. The generation of reactive oxygen species (ROS) was determined by electron spin resonance (ESR) spectra, which was recorded on a EPR 200-Plus spectrometer.

**1.2 Electrochemical measurements**

Electrochemical measurements were performed on the CHI760E workstation (Chenhua Instruments, China), and the standard three-electrode system included a platinum plate as the counter electrode, a commercial Ag/AgCl electrode as the reference electrode, and a working electrode. The working electrode was prepared as follows: 15 mg of sample was thoroughly mixed with 200 μL isopropanol containing 5% Nafion, and the resulting suspension was carefully loaded on the ITO glass substrate (10 × 25 × 1.1 mm) and dried at 60 ^o^C under vacuum for 1 h. 0.1 M Na_2_SO_4_ aqueous solution was employed as the electrolyte for the photocurrent test while the aqueous solution containing 0.1 M KCl and 0.005 M K_3_[Fe(CN)_6_] was employed as the electrolyte for the electrochemical impedance spectroscopy (EIS) measurement. For Mott-Schottky tests, the perturbation was 5 mV with frequencies of 1000, 2000, and 3000 Hz.

**1.3 The assessment of •OH**

The sample suspension (0.5 mg/mL, 200 μL) was mixed with MB (1 mg/mL, 15 μL) in PBS. The absorbance of MB was recorded by UV-vis spectrophotometer. Therefore, by comparing the absorbance of the mixed solution, the relative content of •OH generated by the photo-catalyst after xenon lamp irradiation (300 W) irradiation for 10 min can be evaluated.

**1.4 The assessment of •O_2_^-^**

100 μL of the sample (1 mg/mL) was mixed with 0.9 mL of NBT (25 μM) solution. Therefore, by comparing the absorbance of the mixed solution, we can evaluate the relative content of •O_2_^-^ generated by the photocatalyst after xenon lamp irradiation (300 W) irradiation for 10 min. The absorbance at 560 nm of the mixed solution was then quantified by UV-vis spectrophotometer.

**1.5 The assessment of H_2_O_2_**

10 mg of COFs was suspended in water. Before illumination, the suspension was bubbled with O_2_ for 30 mins to ensure O_2_ saturation. After one hour of reaction, 1 mL of solution was extracted. Then, the concentration of H_2_O_2_ was determined by adding 1 mL of 0.1 M potassium hydrogen phthalate (C_8_H_5_KO_4_) aqueous solution and 1 mL of 0.4 M potassium iodide (KI) aqueous solution. In acidic conditions, the H_2_O_2_ generated oxidized the iodide anions to triiodide anions. This resulted in a strong absorption peak at around 350 nm, which was observable with an UV-vis spectrophotometer.

**1.6 In Vitro Antibacterial Study**

Gram-positive *S. aureus* (ATCC 25923), *MRSA*, and Gram-negative *E. coli* (ATCC 25922) were used as model bacteria. The in vitro antibacterial effect of various COFs was assessed by the plate counting method. Briefly, bacterial dispersion was diluted to 10^6^ CFU/mL with phosphate buffer (PBS, pH 7.4), and subsequently, 0.5 mL of the diluted bacterial suspension was mixed with 0.5 mL of NQ-COF_S1_, NQ-COF_A1_-Me and NQ-COF_S1_-Me (0, 128, 256, 512 µg/mL). After being treated with xenon lamp irradiation (300 W), the mixture solutions were incubated for another 2 h. Finally, the resulting bacterial suspension was diluted with sterile PBS to 10^4^ CFU/mL, and then 40 µL of the diluted bacterial suspension was spread on the solid medium and incubated at 37 °C overnight to count the number of colonies. The inhibition efficiency was determined from the following equation:

Inhibition rate (%)=(1 − CFU_sample_/CFU_Blank_)×100

**1.7 In Vitro Live/Dead Bacterial Cell Staining**

The live/dead bacteria with fluorescent labeling were imaged using a confocal laser scanning microscope (CLSM). Briefly, 500 µL of the COFs - bacterial suspensions (10^6^ CFU/mL) after various treatments were treated with 10 µL of SYTO-9/PI double stain kit including propidium iodide (PI, 4.5 µm) and SYTO 9 (4 µM) in the dark for 30 min. In this process, live bacterial cells were stained into green fluorescence with SYTO-9, and dead bacterial cells were stained into red fluorescence with PI. The resulting bacteria samples were placed on a glass slide surface and observed by CLSM.

**1.8 Catalytic degradation of CAP**

The reaction solution (10 mL) containing 100 μg CAP and 500 μg catalyst was subjected to stirring in darkness for 15 min to attain adsorption/desorption equilibrium, followed by light irradiation using a 300 W xenon lamp. At required intervals, the reaction solution (500 μL) was sampled and filtered through a 0.22 μm membrane to separate the solid catalyst. The residual concentration of pollutant was determined using a high-performance liquid chromatography (HPLC) system.

**1.9 Statistical Analysis**

The error bars in the graph represent the standard deviation from three replicates (n = 3). The p-values were calculated using two-sided One-way ANOVA post-Dunnett’s test. The limit of detection (LOD) was calculated based on the calibration curve with 3S/M, where S is the value of the standard deviation of blank samples, and M is the slope of the standard curve within a low concentration range. GraphPad Prism (version 9.0) and Origin (version 2018) were employed to generate figures and heat maps and perform statistical analyses.

**2. Supplementary Figures**


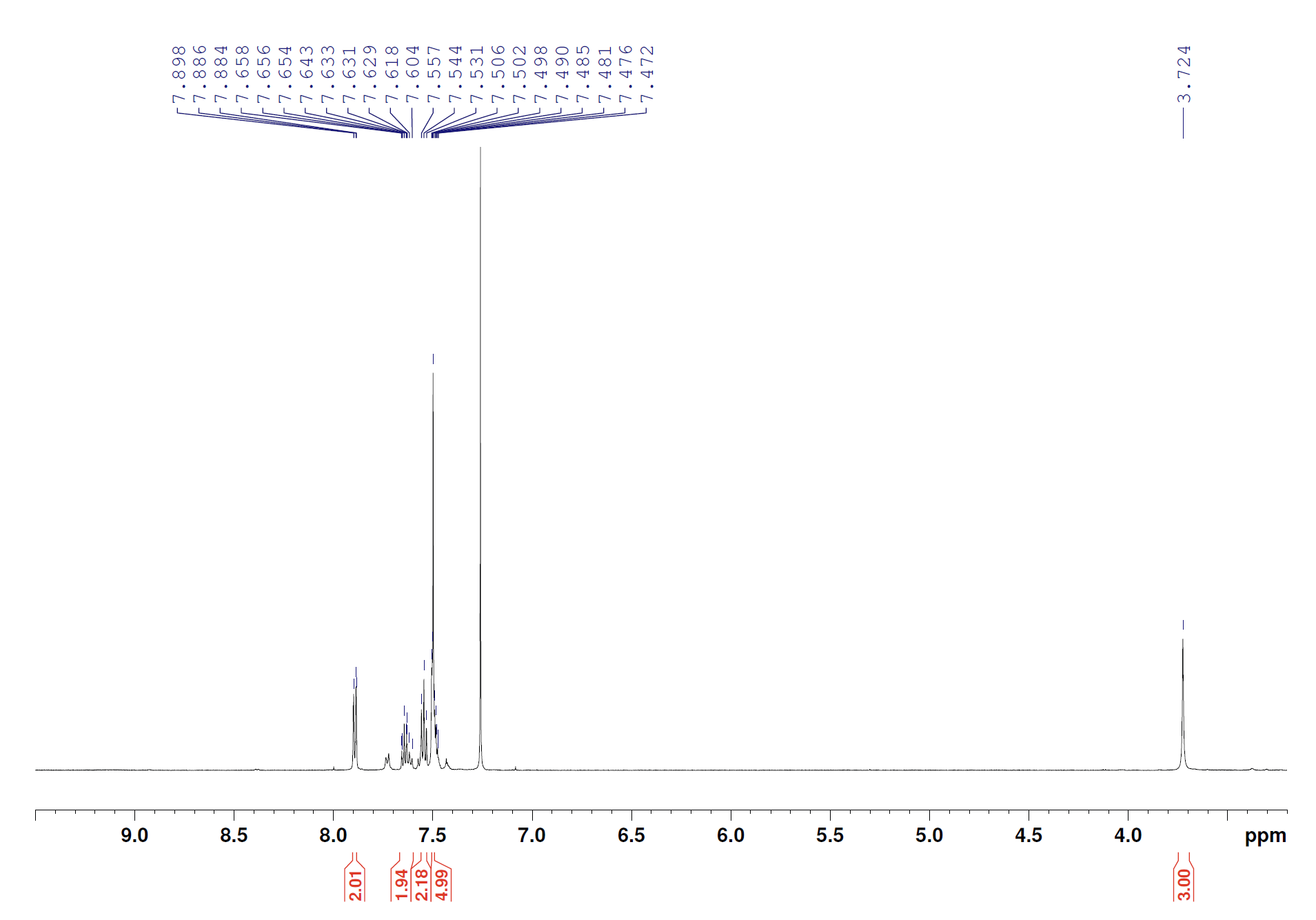


**Figure S1.** NMR spectra of 1-methyl-2-phenylquinolinium.


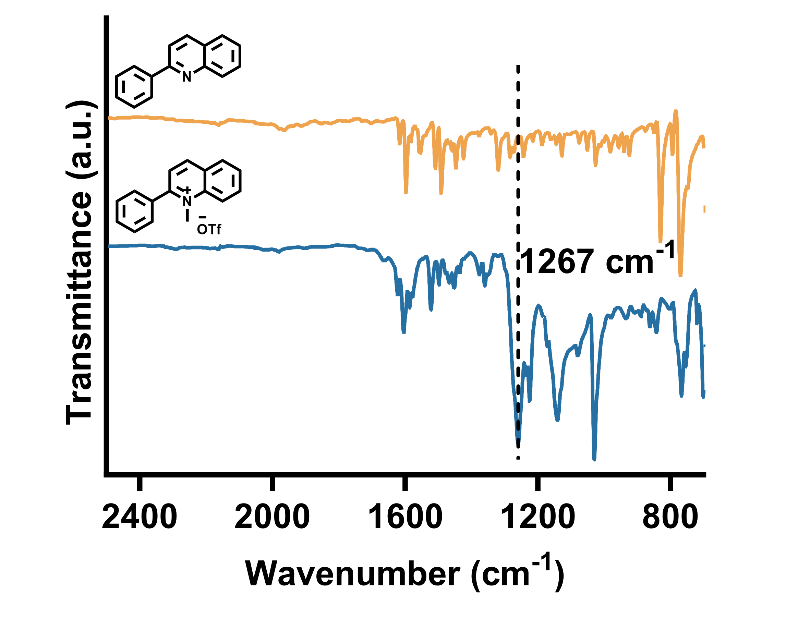


**Figure S2.** FT-IR spectra of 2-phenylquinoline and 1-methyl-2-phenylquinolinium.

**
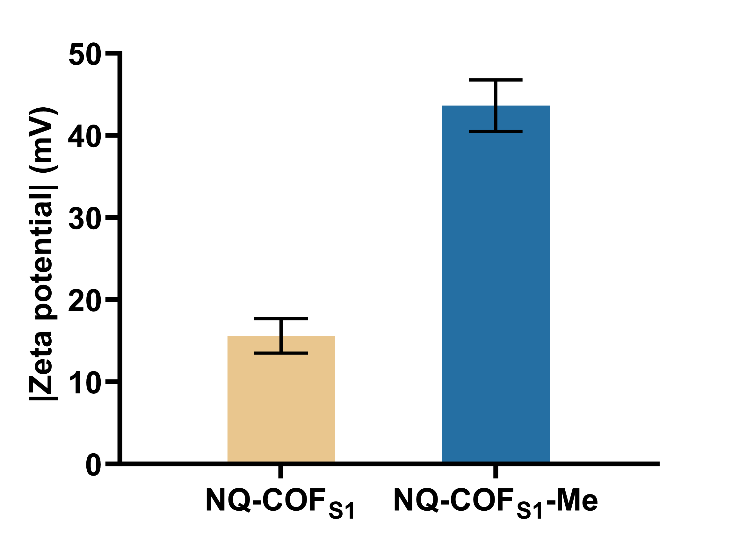
**

**Figure S3.** The absolute values of zeta potential of NQ-COF_S1_ and NQ-COF_S1_-Me.

**
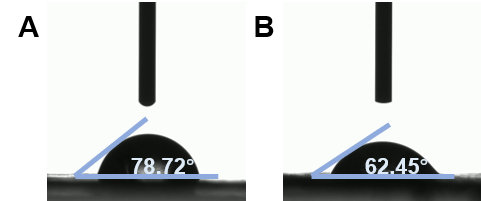
**

**Figure S4.** The water contact angle of NQ-COF_S1_-Me.

**
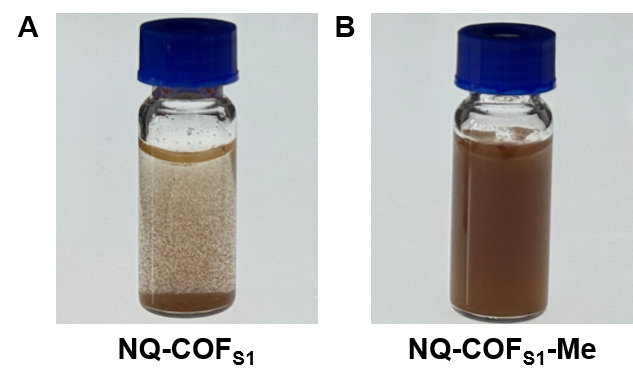
**

**Figure S5.** Actual photograph of NQ-COF_S1_ and NQ-COF_S1_-Me dispersed in water (Without ultrasonic treatment).

**
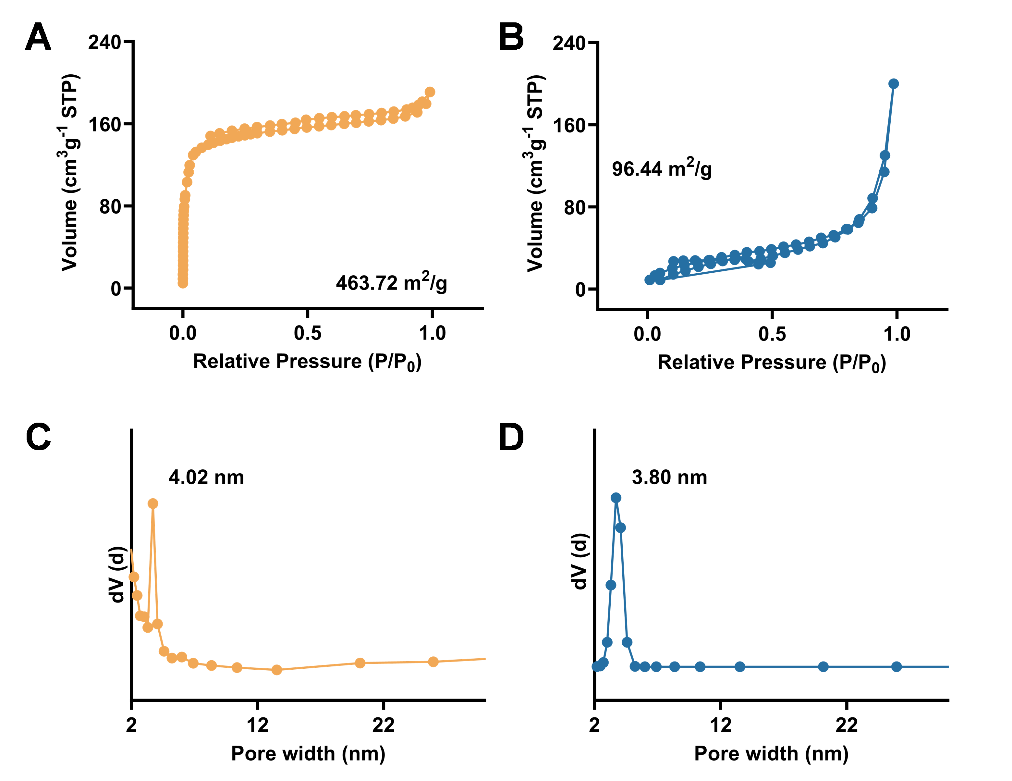
**

**Figure S6.** N_2_ adsorption/desorption isotherms of (A) NQ-COF_S1_ and (B) NQ-COF_S1_-Me. Pore size distributions of (C) NQ-COF_S1_ and (D) NQ-COF_S1_-Me.

**
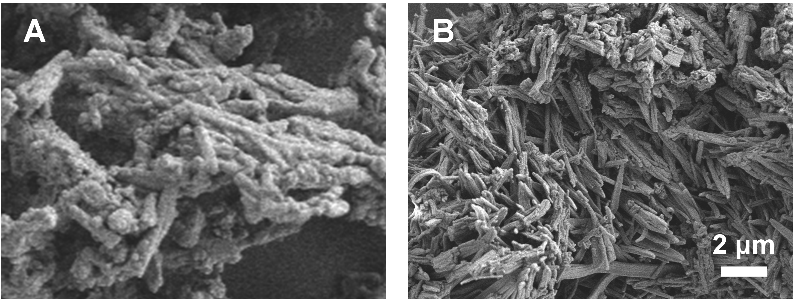
**

**Figure S7.** SEM images of (A) NQ-COF_S1_ and (B) NQ-COF_S1_-Me.

**
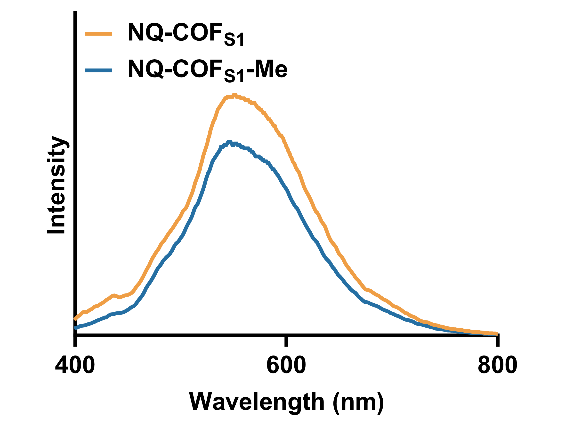
**

**Figure S8.** Steady-state PL spectra of NQ-COF_S1_ and NQ-COF_S1_-Me.

**
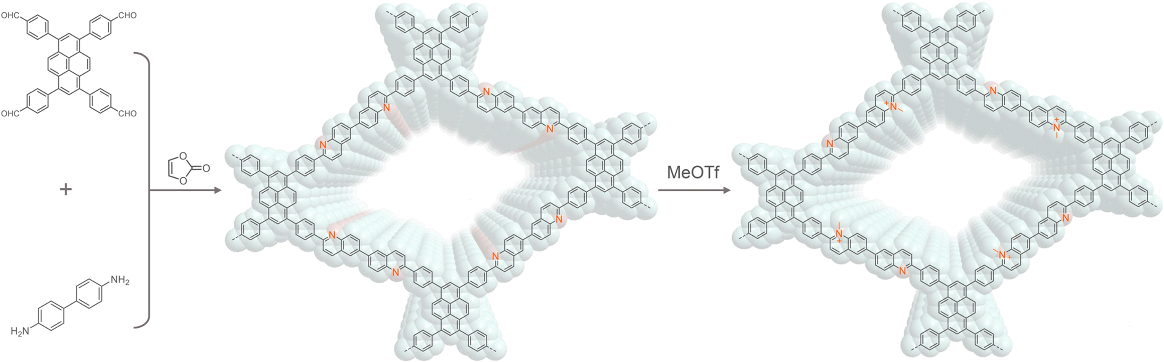
**

**Figure S9.** Schematic illustration of the synthesis of NQ-COF_A1_-Me.

**
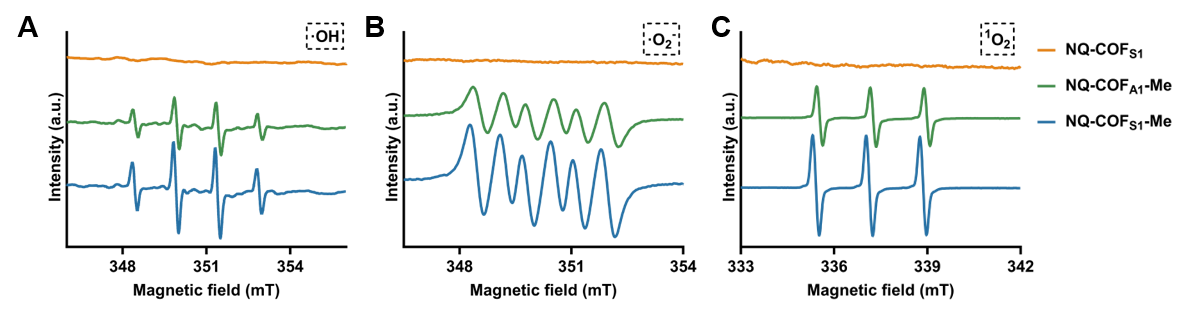
**

**Figure S10.** EPR spectra of (A) •OH, (B) •O_2_^−^ and (C) ^1^O_2_ of NQ-COF_S1_, NQ-COF_A1_-Me and NQ-COF_S1_-Me under irradiation.

**
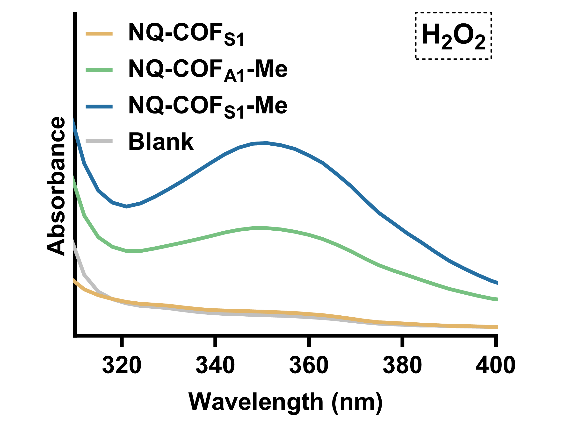
**

**Figure S11.** Absorption spectra of KI-C₈H₅KO₄ probe for H_2_O_2_ generation induced by different COF.

**
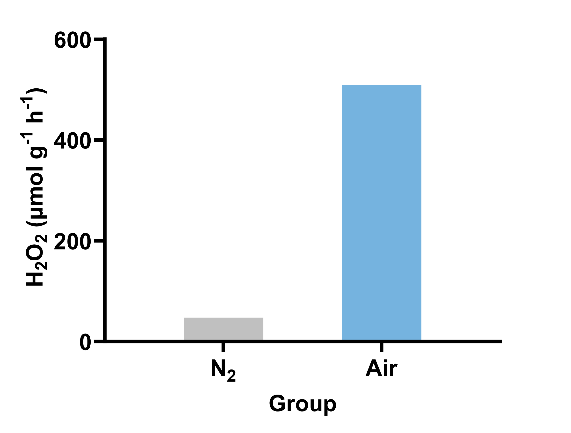
**

**Figure S12.** The generation of H_2_O_2_ in nitrogen and air environments.


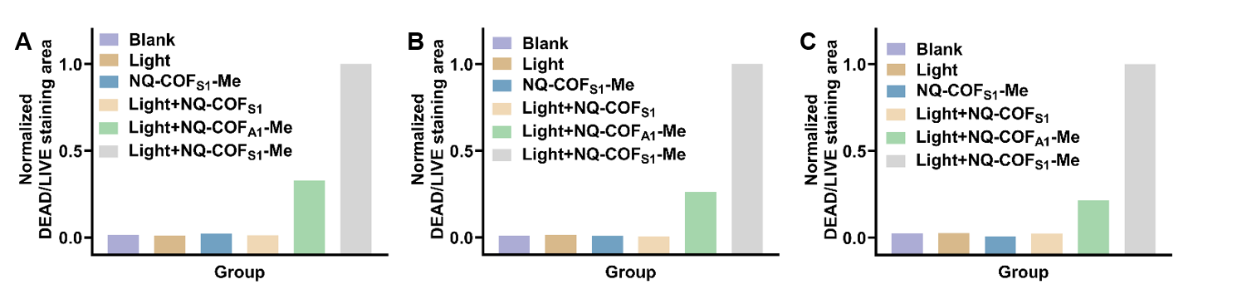


**Figure S13.** The normalized ratio of stained dead to live bacterial areas of different groups of (A) *Salmonella*, (B) *S. aureus* and (C) *MRSA*.

**
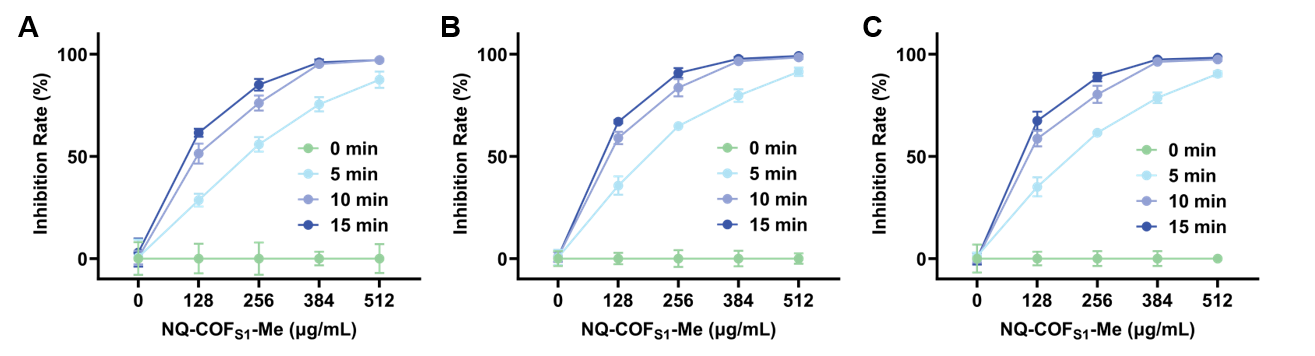
**

**Figure S14.** Inhibition rate of (A) *Salmonella*, (B) *S. aureus* and (C) MRSA by NQ-COF_S1_-Me at different light durations.

**
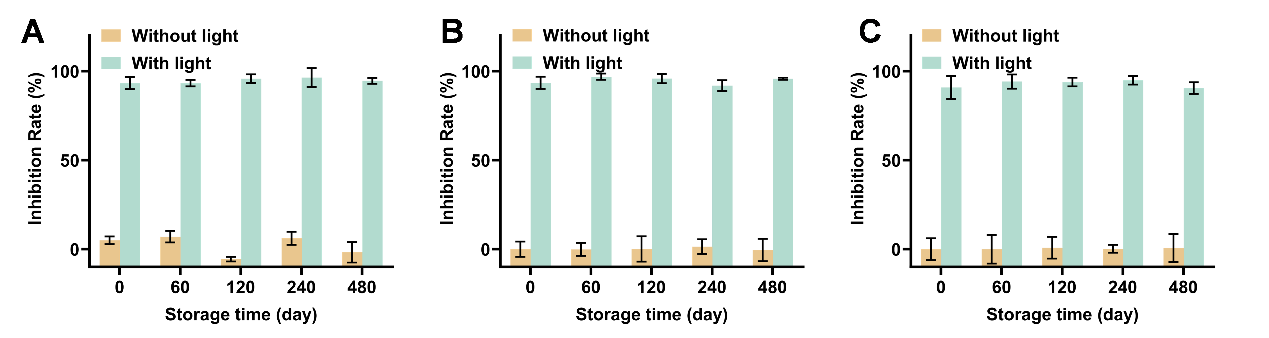
**

**Figure S15.** Inhibition rate of (A) *Salmonella*, (B) *S. aureus* and (C) MRSA by NQ-COF_S1_-Me at different days of storage.

**
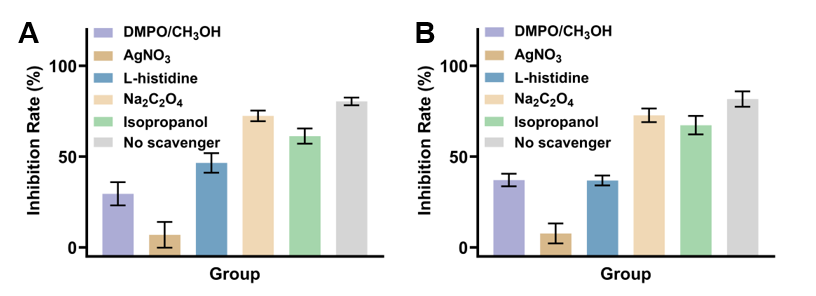
**

**Figure S16.** Effect of scavengers on the performance of NQ-COF_S1_-Me photocatalytic inactivation of (A) *MRSA* and (B) *Salmonella*.

**
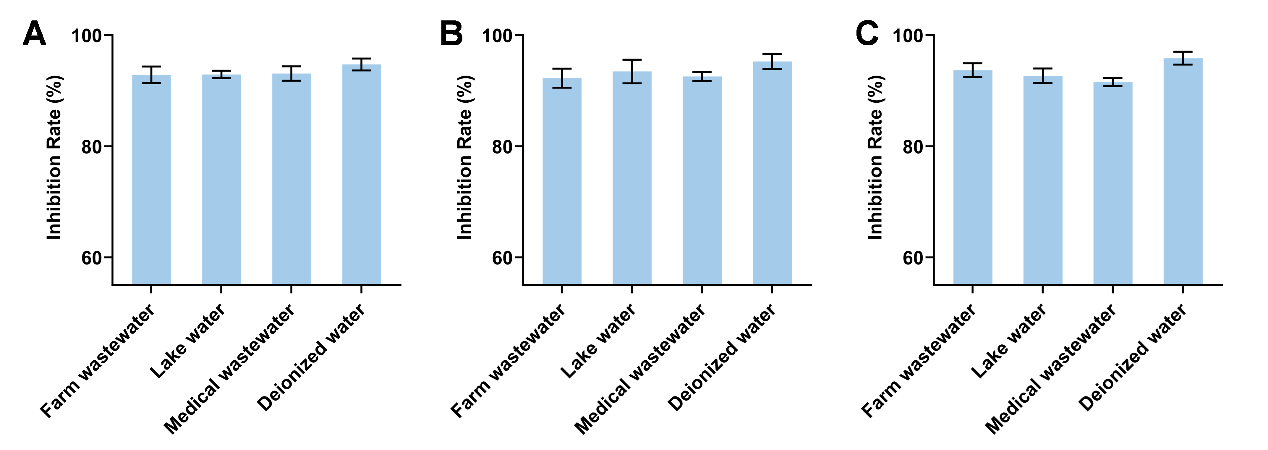
**

**Figure S17.** Antibacterial efficiency of NQ-COF_S1_-Me in different water matrices to (A) *Salmonella*, (B) *S. aureus* and (C) MRSA.

**
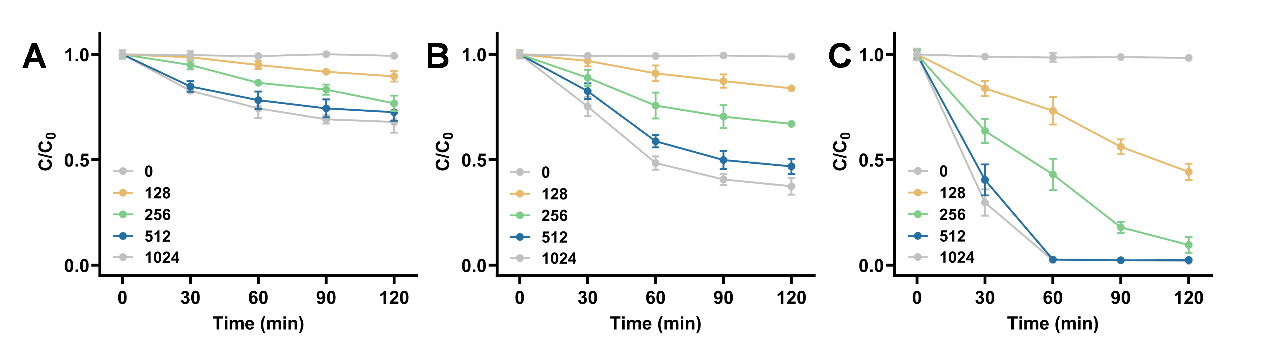
**

**Figure S18.** The degradation of CAP under light irradiation at different concentrations (μg/mL) of (A) NQ-COF_S1_, (B) NQ-COF_A1_-Me and (C) NQ-COF_S1_-Me.

**
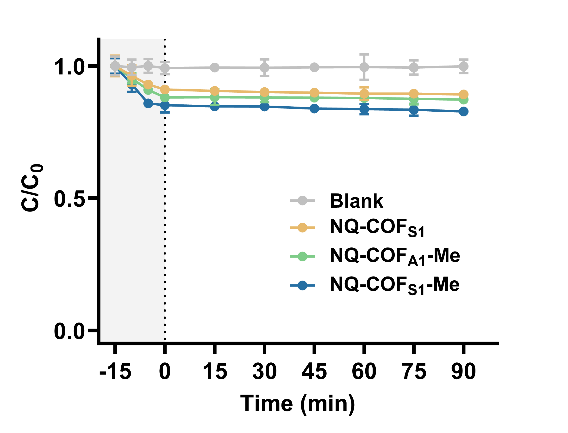
**

**Figure S19.** CAP degradation efficiency under the various COFs treatment without irradiation.


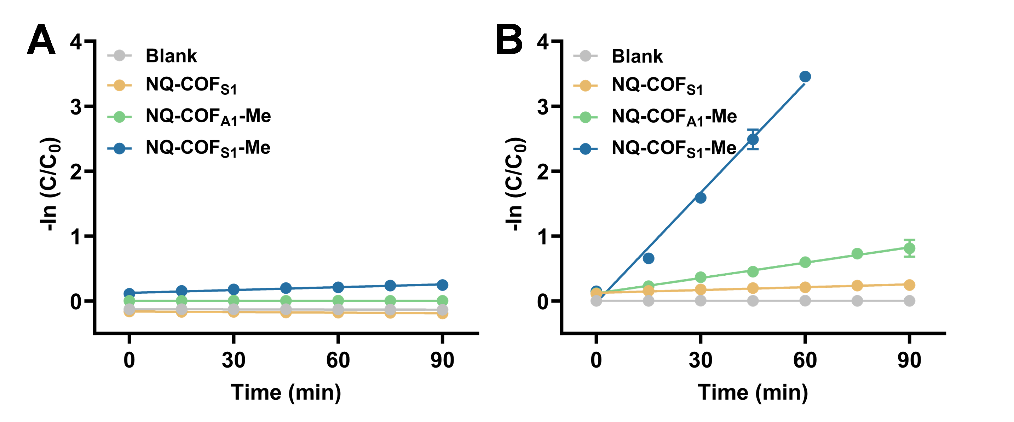


**Figure S20.** Pseudo-first-order kinetic fitting for degradation of CAP (A) in the dark and (B) in light.


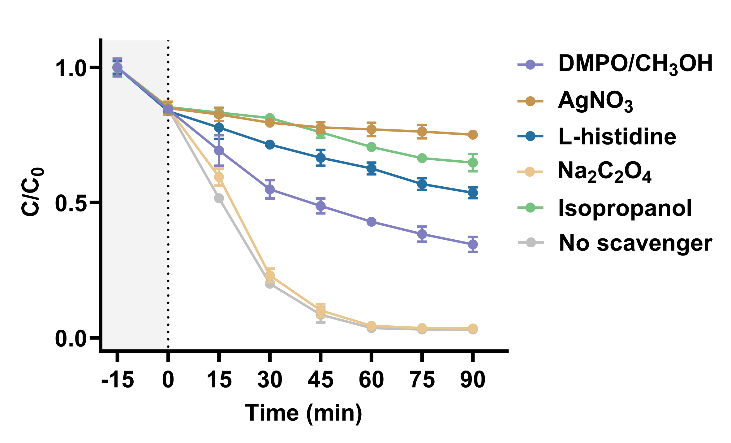


**Figure S21.** Effect of different types of scavengers on CAP removal efficiency.

**
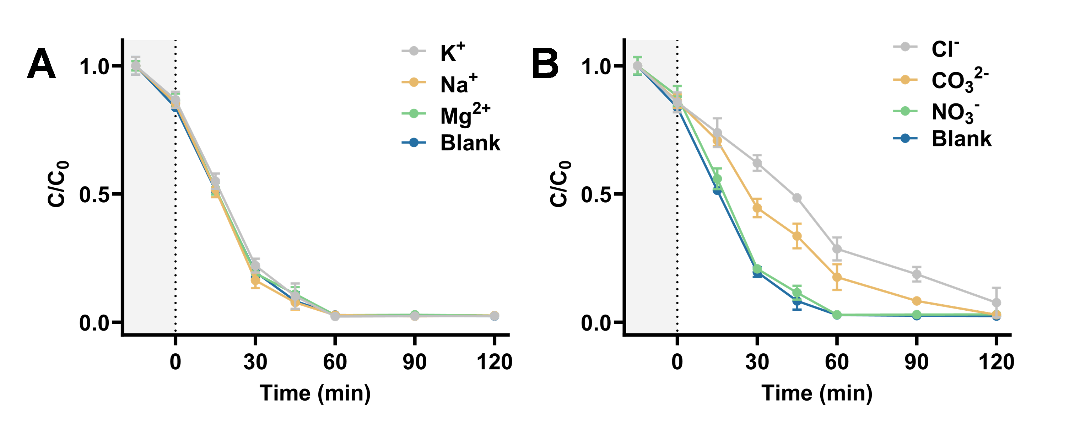
**

**Figure S22.** Effect of different types of (A) cations and (B) anions on CAP removal efficiency.

**
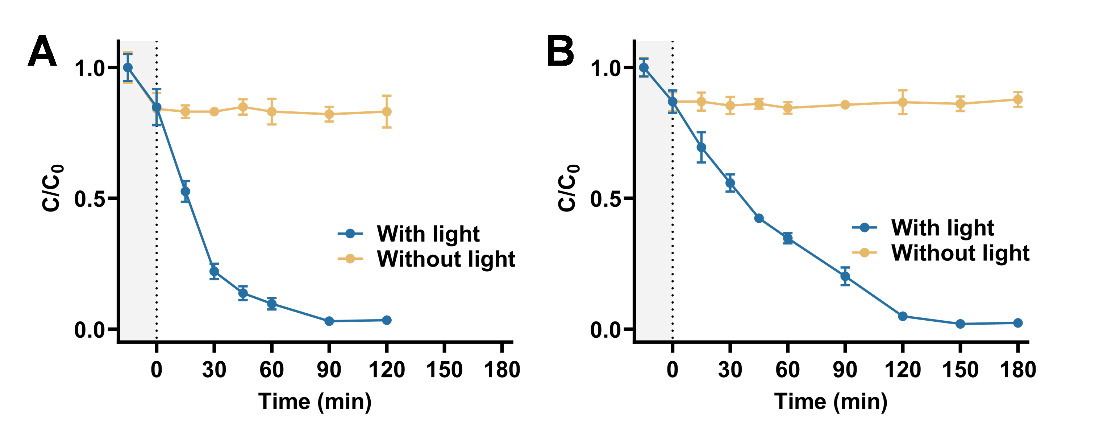
**

**Figure S23.** Performance differences in NQ-COF_S1_-Me for photocatalytic degradation of (A) SMX, (B) NFX.


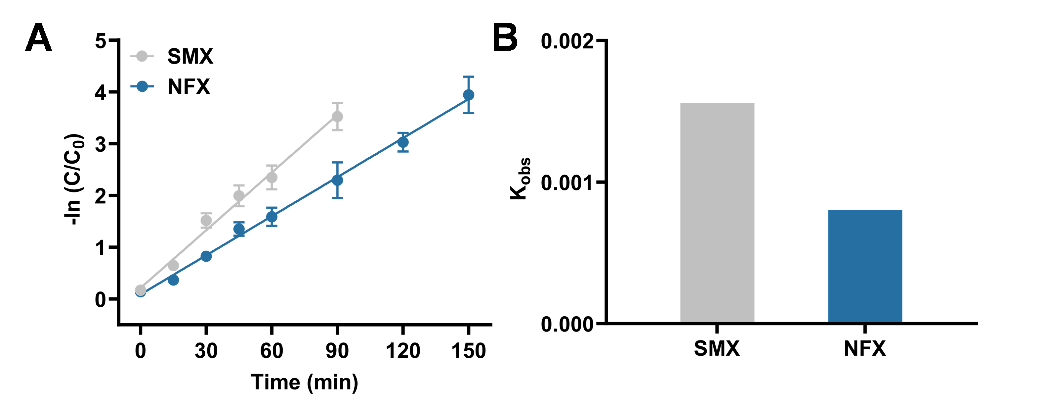


**Figure S24.** (A) Pseudo-first-order kinetic fitting and the (B) corresponding kinetic plots for degradation of SMX, and NFX.
